# An advancement in the synthesis of nano Pd@magnetic amine-Functionalized UiO-66-NH_2_ catalyst for cyanation and O-arylation reactions

**DOI:** 10.1038/s41598-021-90478-y

**Published:** 2021-05-31

**Authors:** Firouz Matloubi Moghaddam, Atefeh Jarahiyan, Mahdi Heidarian Haris, Ali Pourjavadi

**Affiliations:** 1 Laboratory of Organic Synthesis and Natural Products, Department of Chemistry, Sharif University of Technology, Azadi Street, P.O. Box 111559516, Tehran, Iran; 2 Polymer Research Laboratory, Department of Chemistry, Sharif University of Technology, Azadi Street, P.O. Box 111559516, Tehran, Iran

**Keywords:** Chemistry, Nanoscience and technology

## Abstract

The magnetic MOF-based catalytic system has been reported here to be an efficient catalyst for synthesis of benzonitriles and diarylethers of aryl halides under optimal conditions. The MOF catalyst was built based on magnetic nanoparticles and UiO-66-NH_2_ which further modified with 2,4,6-trichloro-1,3,5-triazine and 5-phenyl tetrazole at the same time and the catalyst structure was confirmed by various techniques. This new modification has been applied to increase anchoring palladium into the support. Furthermore, the products’ yields were obtained in good to excellent for all reactions under mild conditions which result from superior activity of the synthesized heterogeneous catalyst containing palladium. Also, the magnetic property of the MOF-based catalyst makes it easy to separate from reaction mediums and reuse in the next runs.

## Introduction

Metal–organic frameworks (MOFs), also called porous coordination polymers or porous coordination networks, are constructed from rigid multipodal organic linkers and inorganic nodes coordination bonds^1^. This very important class of hybrid materials has been utilized in diverse applications for heterogeneous catalysis^2^, adsorption/separation, gas storage, carbon dioxide capture^3,4^, chemical sensors^5^, and drug delivery^6^. It is well-known that a wide variety of MOF applications results from their unique properties, such as high amount of transition metals, adjustable and permanent porosity, ultrahigh surface area, active-site uniformity, and ability of post-synthetic modification^7,8^. In recent years, MOFs have been applied as sufficient catalysts for various organic reactions in different ways: (1) acting metal nodes and linkers as catalysts, (2) accepting guests with catalytic properties like metal nanoparticles^7,9–15^.

Besides of their advantages, low thermal stability of many MOFs makes them unsuitable candidates at high temperature in various applications especially in catalysis^16^. Zirconium-based MOFs (UiO-66-family), with Zr_6_ nodes and ligands such as 1,4-benzenedicarboxylic acid (H_2_BDC) and 2-amino-1,4-benzenedicarboxylic acid (H_2_BDC-NH_2_), have been investigated in literatures extensively owing to their exceptional chemical stability and structural tunability^17,18^. Huang’s group prepared a Pd@UiO-66-NH_2_ catalyst for tandem oxidation-acetalization reaction. This bifunctional catalyst showed excellent catalytic activity and selectivity^19^. Tangestaninejad et al. designed a Pd@UiO-66-NH_2_ catalyst using a direct anionic exchange method and used it in Suzuki–Miyaura cross-coupling reaction with excellent activity^20^. Gao et al. investigated catalytic performance of UiO-66-NH_2_ for knoevenagel condensation of a carbonyl group with the methylene group^21^. Jie et al. studied the application of amine-functionalized UiO-66 for Suzuki and Heck cross-coupling reactions, the amino group of Zr-based MOFs were postmodified with pyridine-2-carboxaldehyde to immobilize Pd nanoparticles^22^.

There have been many reports about functionalization of known MOFs by post-synthetic modification which offer excellent potentials through immobilization of transition metal nanoparticles for a variety of organic reactions^23–25^. The post-synthetic modification of amine-MOFs is widely applied to improve MOFs’ properties for catalytic applications. The amine-MOFs are functionalized easily to make high nitrogen-containing supports which prevent agglomeration and leaching transition metal nanoparticles such as palladium and platinum etc^26^.

On the other hand, nitriles are important category of materials in natural products, organic compounds, pesticides, and pharmaceuticals^27^. Because, nitriles serve as functional groups which can easily transform into corresponding amines, amides, ketones, aldehydes, and esters^28^. Among nitriles, benzonitriles are more attractive compounds which are useful precursors to versatile derivatives^29^. Moreover, diaryl ethers are another important class of organic compounds which are key intermediates for synthesis of pharmaceutical, agrochemical, and biochemical scaffolds^30,31^. One of their synthetic approaches is copper mediate reactions that need stoichiometric amounts of copper and suffer from remaining copper salts in the products^31–33^. Therefore, a number of magnetic supports have been used to immobilize transition-metals to solve mentioned restrictions^34,35^.

**Figure 1:**
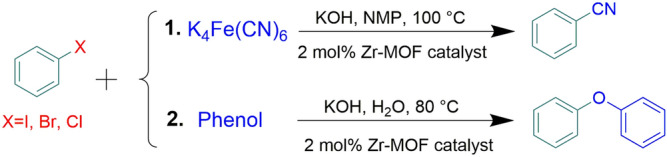
Catalytic activity of Pd^0^@ magnetic amine-Functionalized UiO-66-NH_2_ complex for cyanation and O-arylation.

Inspired by above, we report here Pd-catalyzed cyanation and O-arylation methods which includes designing a magnetic modified UiO-66-NH_2_ as the catalyst support and Pd nanoparticles as the anchored transition-metal. The purpose of UiO-66-NH_2_ modification was preparing nitrogen-rich support by providing NNN pincer-like groups to immobilize Pd nanoparticles into it. This property accompanied by intrinsic porosity of UiO-66-NH_2_ led to high loading of Pd nanoparticles without significant leaching. Finally, the prepared catalyst allows benzonitriles and diaryl ethers formation from aryl halides under mild and simple conditions (Fig. 1).

## Results and discussion

### Catalyst preparation

**Figure 2:**
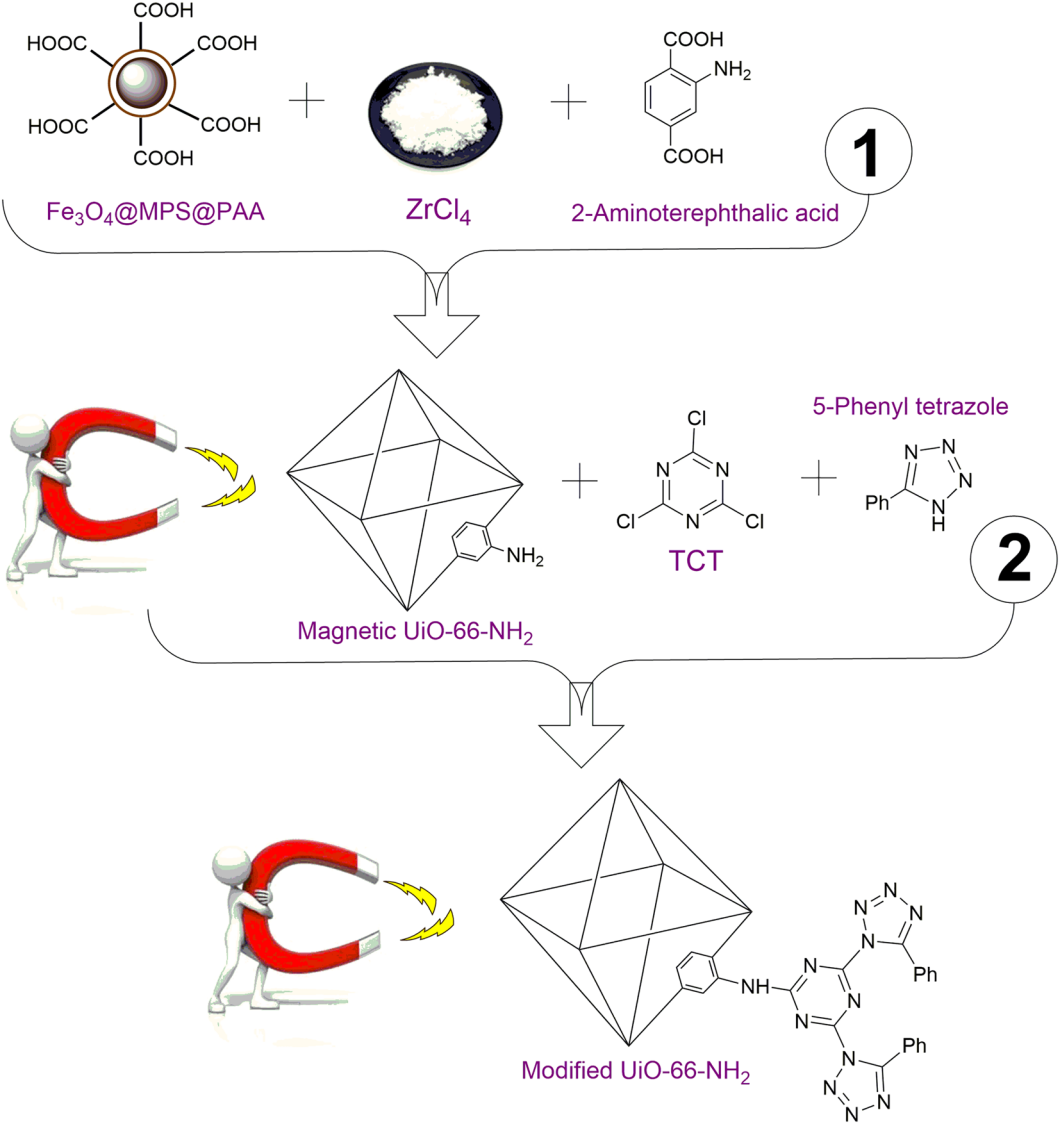
Synthesis procedure of the magnetic amine-Functionalized UiO-66-NH_2_ support.

**Figure 3:**
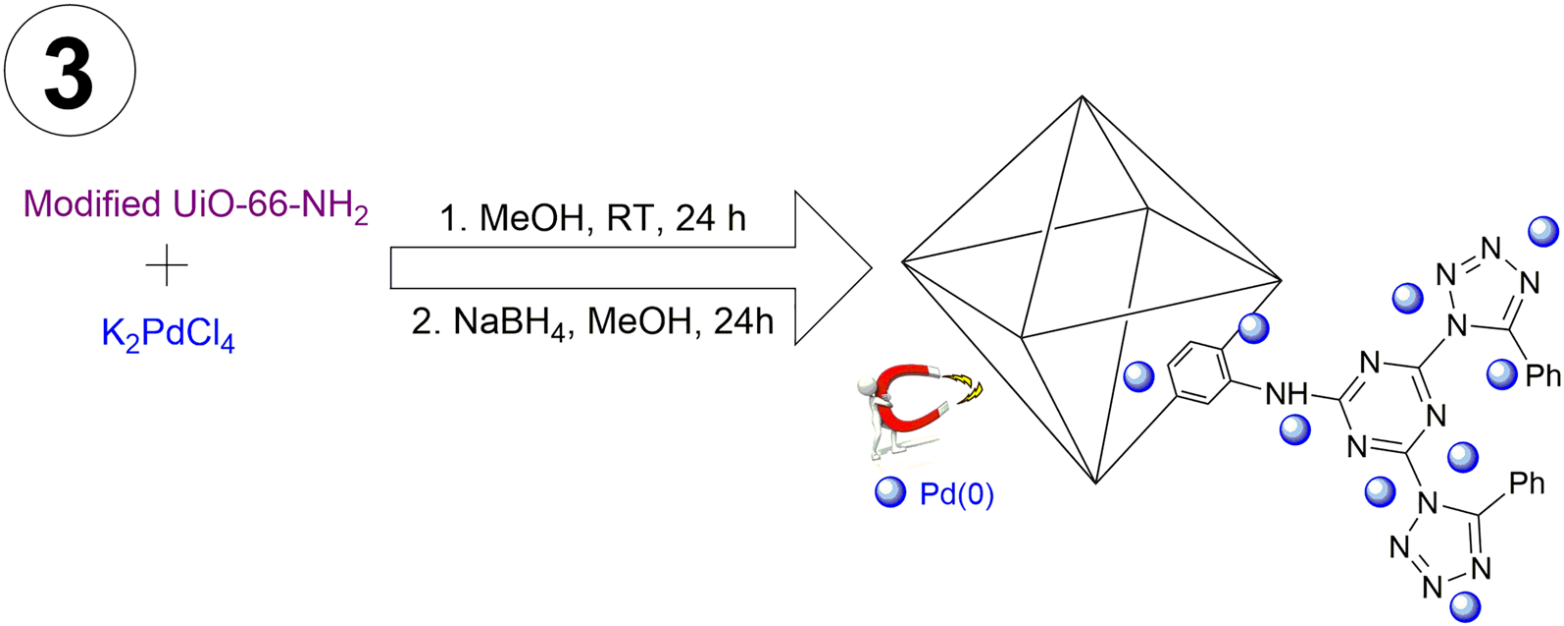
Immobilization of Pd(0) into the magnetic amine-Functionalized UiO-66-NH_2_ support.

The magnetic amine-Functionalized UiO-66-NH_2_, a porous scaffold with large surface area, was synthesized to immobilize palladium nanoparticles. At first, the magnetic nanoparticles were functionalized by acrylic acid (AA) to prepare the surface for growth of UiO-66-NH_2_ (Fig. 2). Subsequently, the amino groups of MOF were modified by 2,4,6-trichloro-1,3,5-triazine (TCT) and 5-phenyl tetrazole which reacted with K_2_PdCl_4_ to provide an efficient heterogeneous catalyst (Fig. 3). Briefly, one chloride of TCT was substituted by amine groups of magnetic UiO-66-NH_2_ support. Then, the remaining two chlorides of TCT were substituted by amine groups of 5-phenyl tetrazole. It was expected, the 3D network structure was formed by substitution of all three chlorides in TCT which could effectively immobilized palladium ions into the resulting support by coordination with NNN pincer-like groups.

### Characterization of Pd/MOF Catalyst

#### FT-IR spectroscopy

**Figure 4:**
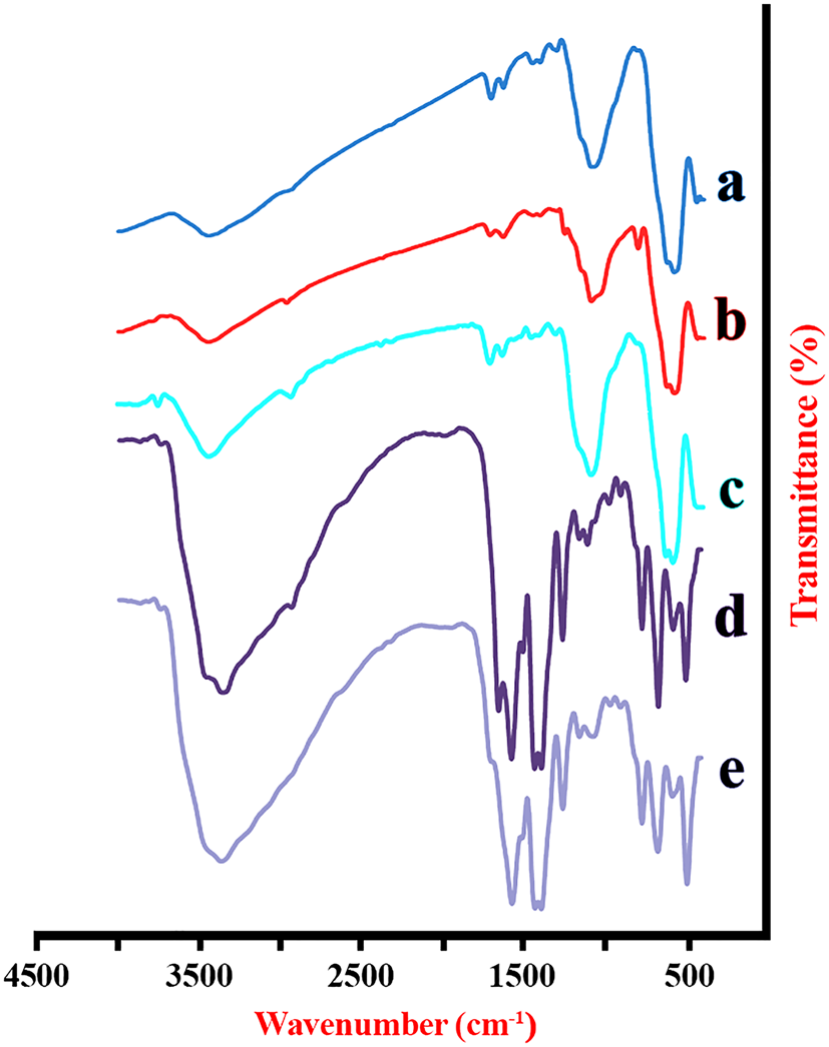
FT-IR spectra of (**a**) Fe_3_O_4_@SiO_2_, (**b**) Fe_3_O_4_@SiO_2_@MPS, (**c**) magnetic PAA, (**d**) magnetic UiO-66-NH_2_ support and (**e**) magnetic amine-Functionalized UiO-66-NH_2_ support.

The characteristic absorption bands at 3440, 1093, and 576 cm^−1^ are related to O-H stretching vibration Si-O and Fe-O stretching vibration, and 3-(trimethoxysilyl)propylmethacrylate (MPS) sharp peaks can be seen at 1714 and 1408 cm^−1^ for C=O and C=C stretching bonds which confirms the Fe_3_O_4_ surface was coated successfully with MPS (Fig. 4)^36^. The PAA illustrates characteristic absorption bands at 2941, 1718, 1460, and 1411 cm^−1^ assigned to the CH_2_ stretching and bending modes, C=O and C–O stretching vibrations in COOH group, respectively^37^. The FT-IR spectrum of magnetic UiO-66 is shown the characteristic vibrational modes including 1573 cm^−1^ for COO− asymmetric mode, 1390 and 1433 cm^−1^ for COO− symmetric modes, 3361 cm^−1^ for NH_2_ symmetric mode, 3467 cm^−1^ for NH_2_ asymmetric mode, and 1259 cm^−1^ for C−N vibrational mode^17^. The magnetic UiO-66-NH_2_ was modified by TCT and 5-phenyl tetrazole at the same time and the strong characteristic band of triazine ring skeleton is observed at around 1570 cm^−1^^38^ and 5-phenyl tetrazole ring show C=N and N=N stretching vibrations at 1450 and 1530 cm^−1^^39^ which these bands overlap with the peaks of the magnetic UiO-66-NH_2_. The peak at 3435 cm^−1^ shows two branches related to stretching mode of NH_2_ groups which transformed to one broad peak related to NH groups after modification. Thus, it is a strong evidence for functionalization of the magnetic UiO-66-NH_2_.

#### XRD patterns

**Figure 5:**
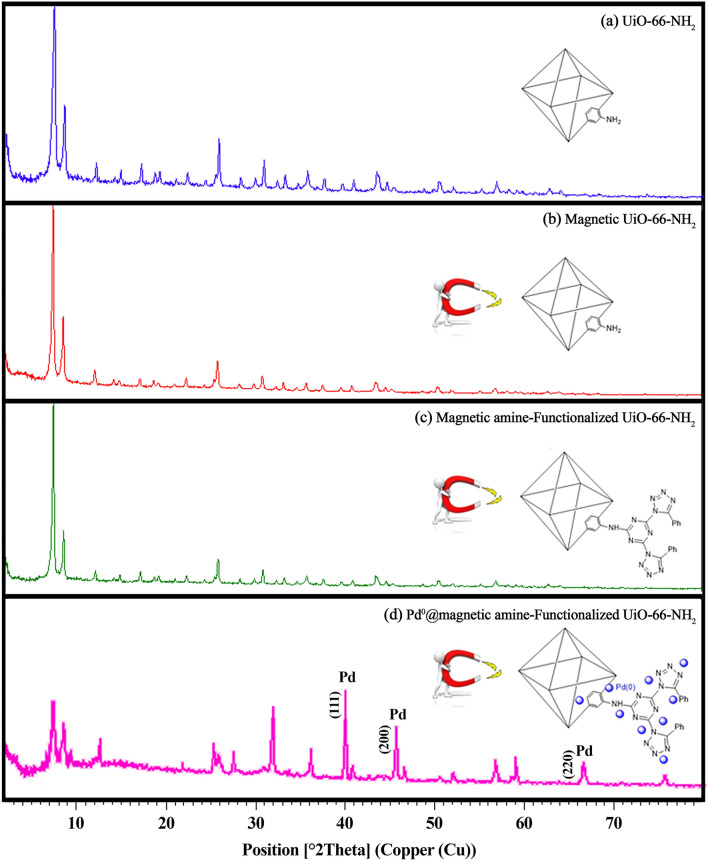
XRD patterns of (**a**) UiO-66-NH_2_, (**b**) magnetic Ui66-NH_2_, (**c**) magnetic amine-Functionalized UiO-66-NH_2_ and (**d**) Pd^0^@magnetic amine-Functionalized UiO-66-NH_2_ catalyst.

XRD patterns of UiO-66-NH_2_, magnetic UiO-66-NH_2_, magnetic amine-Functionalized UiO-66-NH_2_ and Pd^0^@ magnetic amine-Functionalized UiO-66-NH_2_ are presented in Fig. 5. In the patterns, all diffraction peaks are similar to UiO-66 pattern which reported by Wang et al.^16^. These patterns confirm successful synthesis of UiO-66-NH_2_ and the peaks of Fe_3_O_4_ was not apparent in the patterns owing to applying low amount of Fe_3_O_4_ in this procedure. Therefore, the crystalline nature of the magnetic Zr-MOF is preserved after modification. In accordance with Fig. 5d, Pd^0^@ magnetic amine-Functionalized UiO-66-NH_2_ shows the both peaks of UiO-66-NH_2_ and Pd nanoparticles confirming immobilization of Pd nanoparticles into the magnetic amine-Functionalized UiO-66-NH_2_ support.

#### Morphology analysis

**Figure 6:**
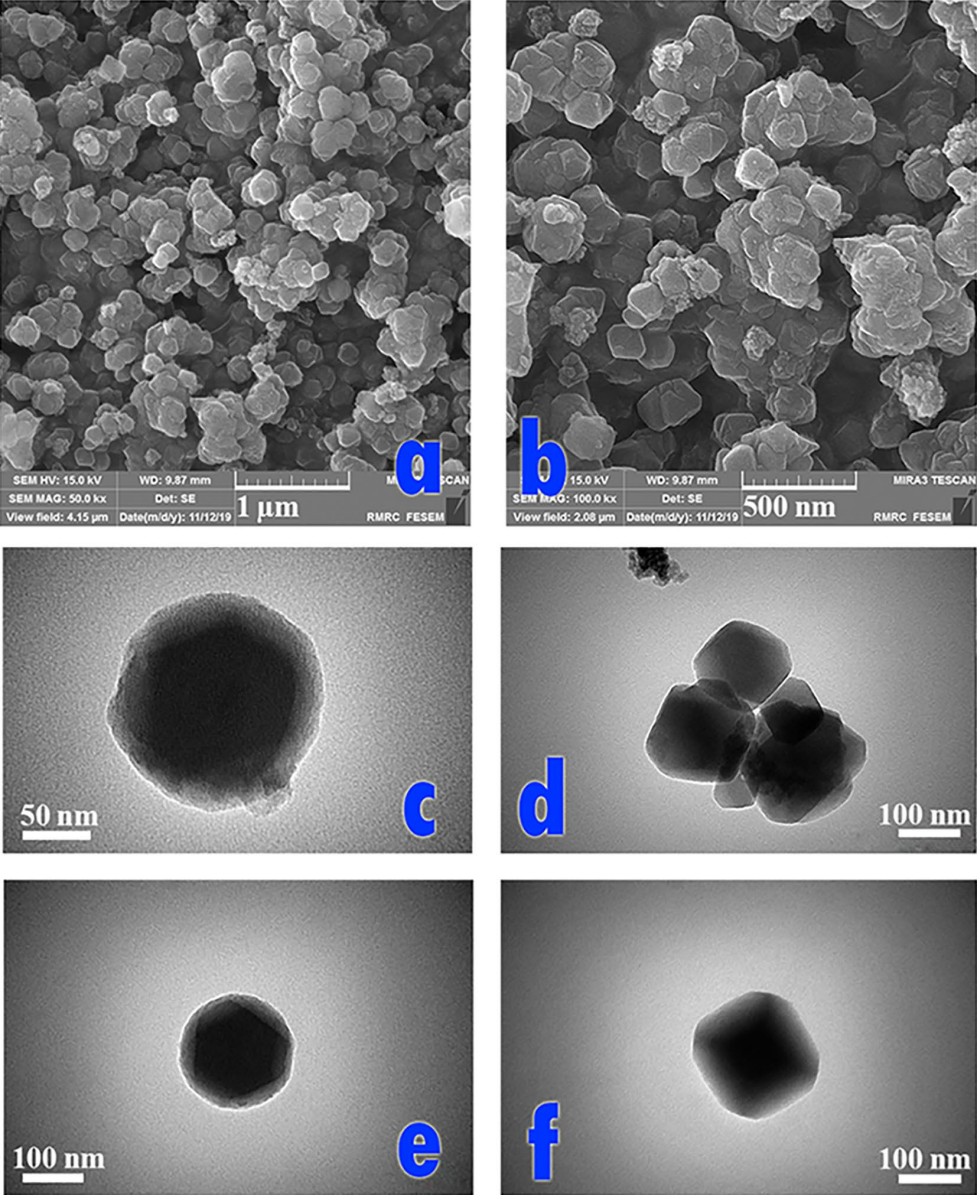
FE-SEM (**a** and **b**) and TEM (**c**–**f**) micrographs of magnetic amine-Functionalized UiO-66-NH_2_ catalyst.

The morphology of the magnetic amine-Functionalized UiO-66-NH_2_ are presented in FE-SEM images (Fig. 6a, b). The FE-SEM images illustrate the particles have a mean diameter 125 nm with cubic structure which are similar to pervious works^17^. In addition, the magnetic amine-Functionalized UiO-66-NH_2_ are uniform without aggregation. TEM images of the prepared MOF show good agreement with other literatures and can confirm the Fe_3_O_4_ core of the obtained UiO-66-NH_2_ (Fig. 6c–f).

**Figure 7:**
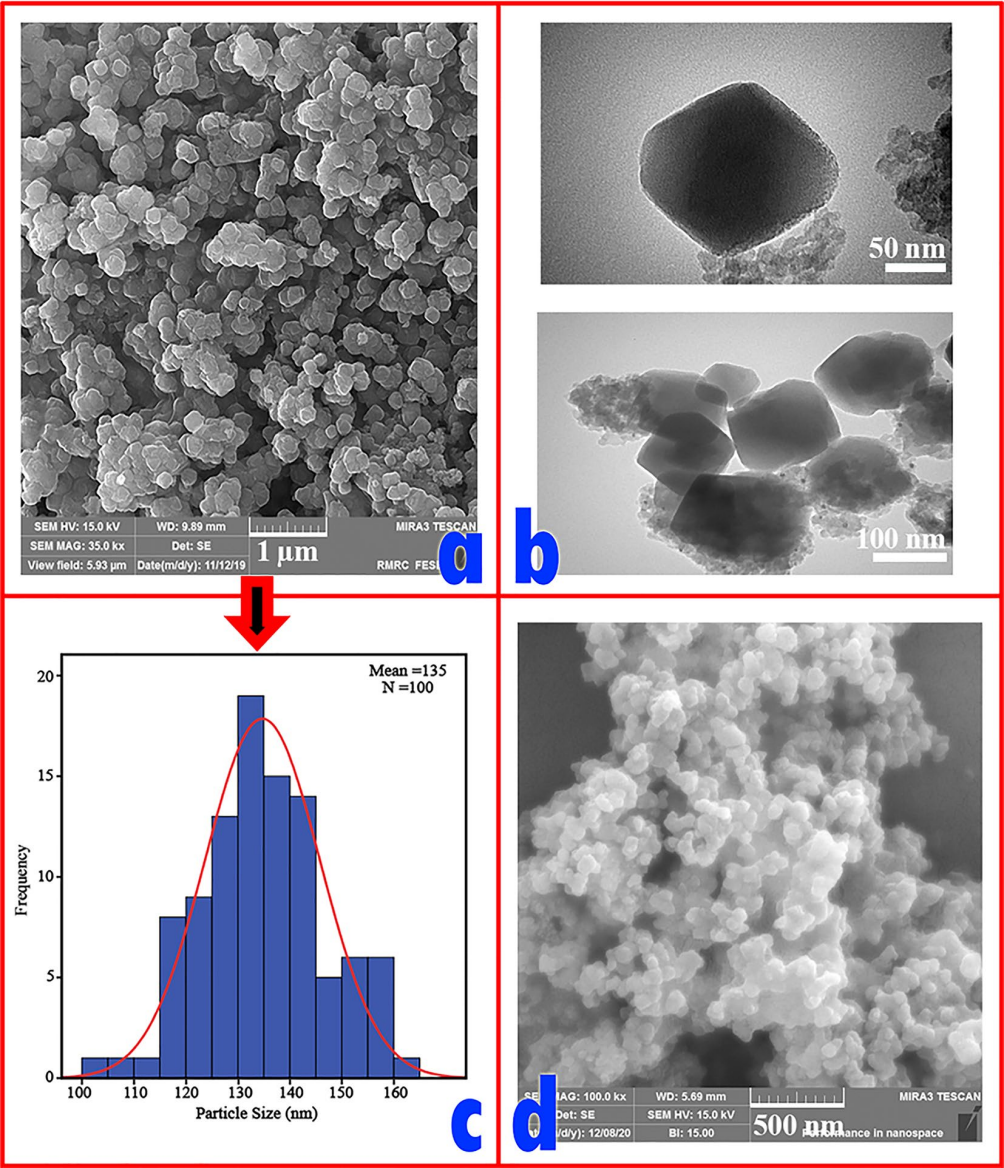
(**a**) FE-SEM and (**b**) TEM micrographs, (**c**) A histogram of Pd^0^@ magnetic amine-Functionalized UiO-66-NH_2_ catalyst and (**d**) A FE-SEM micrograph of recycled Pd^0^@magnetic amine-Functionalized UiO-66-NH_2_ catalyst.

In addition, the FESEM (Fig. 7a) and TEM (Fig. 7b) images of Pd^0^@magnetic amine-Functionalized UiO-66-NH_2_ catalyst are also provided. The size distribution of the catalyst was calculated based on its FESEM image and the mean particle size is around 135 nm (Fig. 7c).

**Figure 8:**
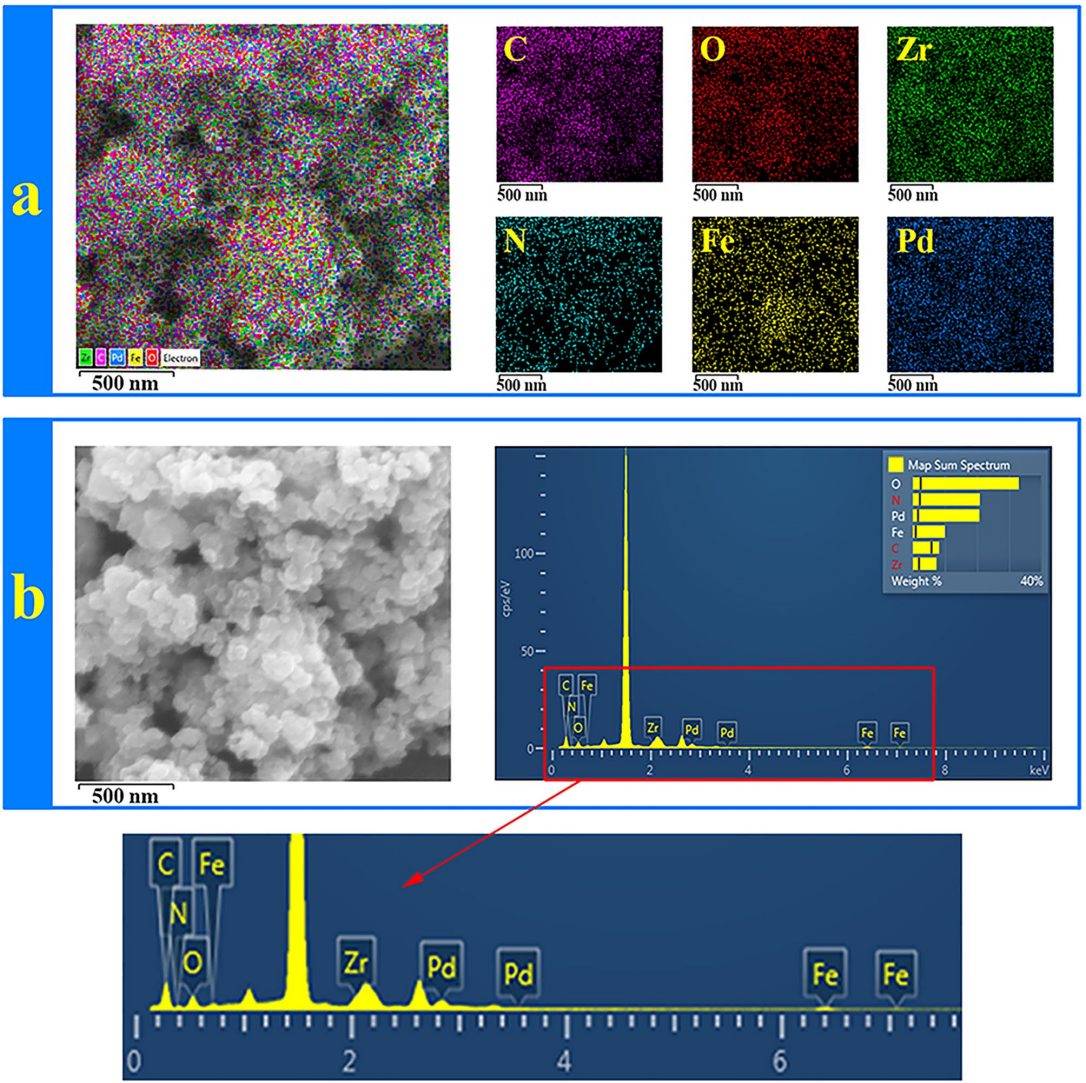
(**a**) Elemental mappings and (**b**) EDX of Pd^0^@ magnetic amine-Functionalized UiO-66-NH_2_ catalyst.

The TEM images of Pd-catalyst have been shown in two magnifications 50 and 100 nm in which the presence of Pd nanoparticles into the polymeric network can be approved (Fig. 7b). The FESEM of recycled Pd^0^@magnetic amine-Functionalized UiO-66-NH_2_ catalyst after six runs for cyanation reaction is presented in Fig. 7d and the small changes in the shape of recycled catalyst are observed. Also, the presence of Pd nanoparticles and other components are confirmed by Elemental mapping and EDX analysis (Fig. 8a, b).

#### Thermal properties

**Figure 9:**
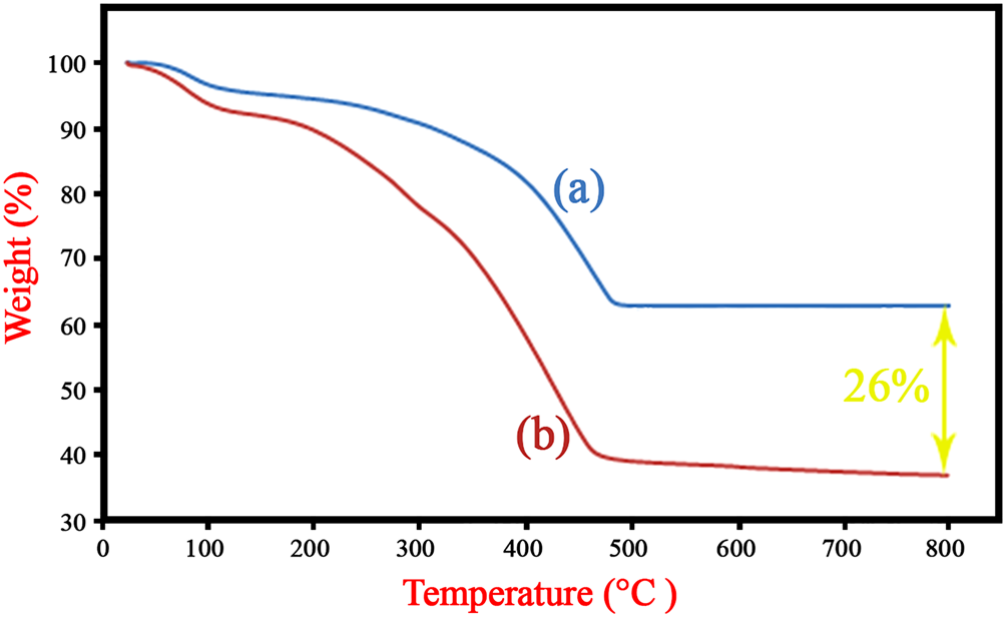
Thermograms of (**a**) magnetic UiO-66-NH_2_ and (**b**) magnetic amine-Functionalized UiO-66-NH_2_ support.

TGA analysis was performed under nitrogen atmosphere at a heating rate 10 °C min^−1^ to investigate thermal decomposition of samples. It is noteworthy, samples were dried overnight in a vacuum oven at 80 °C before analysis. The thermograms of magnetic UiO-66-NH_2_ and magnetic amine-Functionalized UiO-66-NH_2_ are demonstrated in Fig. 9. The first weight loss around 100 °C is related to degradation of water molecules trapping into MOF pores. Furthermore, the second is ascribed to decomposition of organic groups of samples such as H_2_BDC-NH_2_, TCT and 5-phenyl tetrazole. The difference between two curves shows the relative amounts of TCT and 5-phenyl tetrazole grafted to magnetic UiO-66-NH_2_ which is about 26% (w/w).

#### Brunauer–Emmett–Teller (BET) surface area analysis

The N_2_ adsorption–desorption data have been summarized in Table 1. The BET specific surface areas of magnetic amine-Functionalized UiO-66-NH_2_ and Pd^0^@magnetic amine-Functionalized UiO-66-NH_2_ are 828 and 664 m^2 ^g^**−**1^, respectively. In accordance with results, the presence of Pd nanoparticles are confirmed by decreasing BET specific surface area, total pure volume, and mean pore diameter data of Pd^0^@magnetic amine-Functionalized UiO-66-NH_2_ compared with magnetic amine-Functionalized UiO-66-NH_2_^40–42^.

**Table 1:** N_2_ adsorption–desorption data.

Samples	S_BET_ (m^2 ^g^−1^)	Total pure volume (cm^3^ g^−1^)	Mean pore diameter (nm)
Magnetic amine-Functionalized UiO-66-NH_2_	828	0.41	5.1
Pd^0^@magnetic amine-Functionalized UiO-66-NH_2_	664	0.33	2.5

### Cynation over Pd/MOF catalyst

The catalytic activity of the MOF-based catalyst was investigated through C–CN coupling reaction, after conformation of the catalyst structure with some techniques. To achieve optimal conditions, different reaction parameters were screened involving various amounts of catalyst, bases, and solvents by bromobenzene and K_4_Fe(CN)_6_ as a green cyanide source (Table 2). At first, bases including K_2_CO_3_, DABCO, KOH, KHCO_3_, and Et_3_N were tested in the presence of 2.0 mg of MOF based-catalyst and it was found that K_2_CO_3_ and KOH can facilitate the reaction among them but the KOH was the best base and chosen for the reaction (Table 2, entry 1–5). Subsequently, various solvents such as NMP, H_2_O, EtOH, DMSO, DMF, and Toluene were used and the effect of solvents was investigated on the reaction conversion (Table 2, entry 5–10). Based on solvent screening, polar aprotic solvents such as NMP and DMF were more effective rather than polar protic solvents such as H_2_O (Table 2, entry 6), and NMP was more favored solvent (Table 2, entry 5). Then, the reaction was performed with 1.0, 2.0, 5.0 mg of MOF based-catalyst and catalyst without Pd nanoparticles under the same reaction conditions and also the model reaction was tested in the presence of 2.0 mg of Pd^0^@magnetic UiO-66-NH_2_ and desired product was obtained in 62% yield (Table 2, entry 17) compared with Pd^0^@magnetic amine-Functionalized UiO-66-NH_2_ (88% yield). It was found that 2.0 mg% of MOF based-catalyst was the best choice with 88% yield of desired product: aryl halide (1.0 mmol), potassium hexacyanoferrate(II) (0.2 mmol), MOF based-catalyst (2.0 mg) and KOH (1.2 mmol) in NMP at 100 °C (Table 2, entry 5). The turnover frequency (TOF) value of the catalyst was calculated 3.13 s^−1^ for this model reaction under optimazation conditions.

**Table 2:** Variation of reaction conditions for the cyanation of bromobenzene^a^.


Entry	Catalyst (mg)	Base	Solvent	Temp (°C)	Isolated yield (%)
1	2	K_2_CO_3_	NMP	100	77
2	2	DABCO	NMP	100	Trace
3	2	KHCO_3_	NMP	100	27
4	2	Et_3_N	NMP	100	20
5^b^	2	**KOH**	**NMP**	100	88
6	2	KOH	H_2_O	100	24
7	2	KOH	EtOH	100	29
8	2	KOH	DMSO	100	68
9	2	KOH	DMF	100	83
10	2	KOH	Toluene	100	41
11	1	KOH	NMP	100	66
12	5	KOH	NMP	100	89
13	2	KOH	NMP	80	76
14	2	KOH	NMP	R.T	35
15	2 mg of Cat. without Pd	KOH	NMP	100	22
16	–	KOH	NMP	100	Trace
17	2 mg of Pd^0^@ magnetic UiO-66-NH_2_	KOH	NMP	100	62

^a^Bromobenzene (1.0 mmol), Potassium hexacyanoferrate(II) (0.2 mmol), Base (1.2 mmol), Solvent (2.0 ml) and MOF-based catalyst.

^b^ Optimal parameters.

With having the best reaction parameters in hand, the applicability of the reported protocol was studied for versatile aryl halides bearing both electron-deficient and electron-rich functional groups to provide target products with good to excellent yields. The aryl halides were reacted with K_4_Fe(CN)_6_ in the presence of KOH and 2.0 mg of MOF based-catalyst and NMP under 100 °C as optimal conditions as presented in Table 3. Generally, aryl iodides and aryl bromides with electron-deficient groups such as nitro groups in meta and para positions have shown excellent yields (Table 3, entry 7 and 16) and less yields were seen in products having electron-rich groups such as methyl groups (Table 3, entry 5 and 12). Also, in this case aryl chlorides have demonstrated good yields of benzonitriles which their reactions proceed with longer times. The aryl chloride bearing electron-deficient (Table 3, entry 21–23) have shown higher yields in comparison to electon-rich aryl chloride and they need higher temperature and longer reaction time to provide desired yield (Table 3, entry 24).

**Table 3:** Cyanation of various aryl halides.


Entry	Arylhalide	Temp (°C)/ Time (h)	Product	Isolated yield (%)
1		100/2		92
2		100/2		94
3		100/2		96
4		100/4		89
5		100/4		87
6		100/1		96
7		100/1		98
8		100/2		93
9		100/5		88
10		100/6		83
11		100/6		81
12		100/6		80
13		120/5		81
14		110/5		79
15		100/3		90
16		100/3		91
17		100/3		90
18		100/3		94
19		100/3		91
20		110/7		73
21		100/6		84
22		100/6		88
23		100/6		88
24		120/7		60

Aryl halide (1.0 mmol), Potassium hexacyanoferrate(II) (0.2 mmol), Potassium hydroxide (1.2 mmol), NMP (2.0 ml) and MOF-based catalyst (2.0 mg).

Then, the heterocyclic compounds including 2- and 4-bromopyridine were examined and the 2- cyanopyridine (94%) and 4-cyanopyridine (91%) were obtained in excellent yields (Table 3, entry 18 and 19).

The chemoselectivity of this protocol was tested by 1-chloro-2-iodobenzene and 1-chloro-4-iodobenzene which they converted to 2-chlorobenzonitrile and 4-chlorobenzonitrile with excellent yields, 94% and 96%, respectively (Table 3, entry 2 and 3). Moreover,1-bromo-4-iodobenzene and 1-bromo-4-chlorobenzene provided 4-bromobenzonitrile and 4-chlorobenzonitrile, 93% and 90%, respectively (Table 3, entry 8 and 17). Therefore, the reactions indicated excellent chemoselectivity which occurred at iodine and bromine positions as better leaving groups (See Supplementary information for ^1^H and ^13^C NMR spectral data).

### O-arylation over Pd/MOF catalyst

In the following, the catalytic activity of the MOF based-catalyst has been investigated for O-arylation of phenols. The reaction conditions have been optimized for the phenol and bromobenzene as partner substrates and the data is summarized in Table 4. In accordance with Table 4, various bases such as K_2_CO_3_, K_3_PO_4_, KOH, NaHCO_3_, and Et_3_N were applied and the KOH was the best base (Table 4, entry 1–5). In the next step, solvents were screened and DMSO and H_2_O were the optimal solvents rather than DMF, NMP, CH_3_CN, and Toluene (Table 4, entry 3 and entry 6–10). But water was chosen as the optimal solvent because of its green nature. Afterwards, the reaction was tested at room temperature, 80 °C and 120 °C (Table 4, entry 11–13). When the reaction took place at 80 °C instead of 100 °C, the desired product was acquired without remarkable change in the yield. To optimize amounts of the catalyst, different amounts of the MOF based-catalyst was used (1.0, 2.0, 5.0 mg, and none) and catalyst without Pd nanoparticles which the 2.0 mg of the catalyst was gave the best yield and use of 5.0 mg of the catalyst showed no significant effect on the yield (Table 4, entry 14–17). Also, the model reaction was tested in the presence of 2.0 mg of Pd^0^@magnetic UiO-66-NH_2_ and desired product was obtained in 67% yield (Table 4, entry 18) compared with Pd^0^@magnetic amine-Functionalized UiO-66-NH_2_ (92% yield). Finally, the optimal conditions were obtained as follows: aryl halide (1.0 mmol), phenol (1.0 mmol), MOF based-catalyst (2.0 mg), KOH (1.2 mmol) in H_2_O at 80 °C (Table 4, entry 11). The TOF value of the catalyst was calculated 5.46 s^−1^ for this model reaction under optimazation conditions.

**Table 4:** Optimization condition for Nano Pd^0^@magnetic amine-Functionalized UiO-66-NH_2_ catalyst on C–O bond formation^a^.


Entry	Catalyst (mg)	Base	Solvent	Temperature (°C)	Isolated yield (%)
1	2	K_2_CO_3_	DMSO	100	88
2	2	K_3_PO_4_	DMSO	100	86
3	2	KOH	DMSO	100	93
4	2	NaHCO_3_	DMSO	100	73
5	2	NEt_3_	DMSO	100	54
6	2	KOH	DMF	100	81
7	2	KOH	NMP	100	73
8	2	KOH	CH_3_CN	100	77
9	2	KOH	H_2_O	100	93
10	2	KOH	Toluene	100	47
11^b^	2	**KOH**	**H**_**2**_**O**	80	92
12	2	KOH	H_2_O	120	93
13	2	KOH	H_2_O	R.T	25
14	5	KOH	H_2_O	80	92
15	1	KOH	H_2_O	80	79
16	2 mg of Cat. without Pd	KOH	H_2_O	80	28
17	–	KOH	H_2_O	80	Trace
18	2 mg of Pd^0^@ magnetic UiO-66-NH_2_	KOH	H_2_O	80	67

^a^Bromobenzene(1.0 mmol), Phenol (1.0 mmol), Base (1.2 mmol), Solvent (2.0 ml) and MOF-based catalyst.

^b^ Optimal parameters

With optimal conditions in hand, we have synthesized a number of diaryl ether derivatives with phenol (1.0 mmol) and aryl halide (1.0 mmol) under the MOF based-catalyst (2.0 mg) and KOH (1.2 mmol) in H_2_O at 80 °C (Table 5). To gain different O-arylated derivatives, several electron-rich and electron-deficient substrates were tested. Firstly, the scope of aryl halides was studied and Iodobenzene and bromobenzene illustrated higher activity in comparison with chlorobenzene because of lower polarizability of C–Cl bond related to oxidation-addition step of palladium insertion in reaction mechanism (Table 5). Subsequently, the electron-rich aryl halides such as methyl and methoxy groups on them showed good yields and electron-deficient aryl halides showed higher yields. In the next step, phenols were examined and electron-rich phenols with methyl and methoxy groups were provided diaryl ethers in excellent yields and electron-deficient phenols having nitro groups depicted lower yields because of decreasing the nucleophilicity of phenols. In the case of 2-nitrophenol which has greater steric hindrance compared with 4-nitrophenol, it was provided the expected products but in low yields. Also, 1-naphthol and 2-naphthol were tested and they generated the desired products in good yields under longer reaction times (See Supplementary information for ^1^H and ^13^C NMR spectral data).

**Table 5:** Reaction scope of nano Pd^0^@magnetic amine-Functionalized UiO-66-NH_2_ catalyst on diarylether formation^a^.


			
X = I	Yield = 95%						
X = Br	Yield = 92%	X = Br	Yield = 91%	X = Br	Yield = 89%	X = Br	Yield = 88%
X = Cl	Yield = 84%						
			
						X = I	Yield = 97%
X = Br	Yield = 86%	X = Br	Yield = 80%	X = Br	Yield = 90%	X = Br	Yield = 94%
						X = Cl	Yield = 89%
			
		X = I	Yield = 95%				
X = Br	Yield = 91%	X = Br	Yield = 93%	X = Br	Yield = 90%	X = Br	Yield = 91%
		X = Cl	Yield = 87%				
			
X = I	Yield = 93%	X = I	Yield = 77%	X = I	Yield = 93%	X = I	Yield = 94%
X = Br	Yield = 90%	X = Br	Yield = 73%	X = Br	Yield = 89%	X = Br	Yield = 89%
X = Cl	Yield = 87%	X = Cl	Yield = 21%	X = Cl	Yield = 81%	X = Cl	Yield = 80%

^a^Aryl halide (1.0 mmol), Phenol (1.0 mmol), Potassium hydroxide (1.2 mmol), H_2_O(2.0 ml) and MOF-based catalyst (2.0 mg).

### Catalyst recycling

The reuseability of the catalyst was examined through optimized reaction conditions between iodobenzene and K_4_Fe(CN)_6_ as model raw materials. After each run, the catalyst was collected by an external magnetic field and the isolated catalyst was washed with methanol and water, dried completely, and applied for next run. This MOF-based catalyst was used over six successive runs and the isolated yields were shown in Table 6. The results confirm that this catalytic system remained still active during six runs of cyanation reaction without loss of catalytic activity. Also, the recycleability of the catalyst was tested for synthesis of diaryl ethers between iodobenzene and phenol as model reaction and the catalyst was reused over five successive runs based on the mentioned procedure (Table 7). After final runs, the loading amounts of Pd were investigated by ICP-OES analysis and they were 0.72 mmol g^−1^ for Cyanation and 0.71 for O-arylation.

**Table 6:** The recyclability of the Pd^0^@magnetic amine-Functionalized UiO-66-NH_2_ in the synthesis of benzonitriles under optimal condition.

Run	1	2	3	4	5	6
Yield%	92	91	91	90	88	83

**Table 7:** The recyclability of the Pd^0^@magnetic amine-Functionalized UiO-66-NH_2_ in the synthesis of diaryl ethers under optimal condition.

Run	1	2	3	4	5
Yield%	95	95	93	91	88

## Conclusion

In summary, the presented article described the preparation and application of the palladium MOF-based catalyst. The magnetic catalyst includes UiO-66-NH_2_ which has been modified with 2,4,6-trichloro-1,3,5-triazine and 5-phenyl tetrazole to support palladium nanoparticles. The shape and morphology of the modified UiO-66-NH_2_ was confirmed by FESEM and TEM analysis and they corresponded with pervious literatures. This catalytic system has shown very efficient activity for both syntheses of cyanoarenes and diaryl ethers in mild reaction conditions with good to excellent yields.

## Experimental section

### Catalyst preparation

#### Preparation of the magnetic nanoparticles

##### Immobilization of acrylic acid on Fe_3_O_4_@SiO_2_ Microspheres

Firstly, the Fe_3_O_4_@SiO_2_ nanoparticles were prepared through co-precipitation method and treated with 3-(trimethoxysilyl)propylmethacrylate (MPS) as reported in previous works^43^. Afterwards, the surface of Fe_3_O_4_@SiO_2_@MPS was functionalized with acrylic acid (AA): 3.0 ml of acrylic acid was added to 0.50 g of Fe_3_O_4_@SiO_2_@MPS in 20 ml deionized water. Then, the flask was charged with 10 mg of AIBN after degassed under N_2_ atmosphere and refluxed for 24h. Then, the obtained magnetite nanoparticles were collected by an external magnet and washed with deionized water/methanol three times, and dried in a vacuum oven at 60 °C for 12h to provide the magnetic PAAs.

#### Preparation of the magnetic UiO-66-NH_2_

The magnetic UiO-66-NH_2_ was synthesized based on literature reported by Wang et al.^16^. In a round bottom flask, 0.2 g of the magnetic PAAs with ZrCl_4_ (2.27 mmol): H_2_BDC-NH_2_ (2.27 mmol): DMF (405.38 mmol) ratio were mixed and put in an autoclave at 120 °C for 24 h. The magnetic amine-functionalized UiO-66 was immersed in chloroform for few days, filtered and dried in a vacuum at 160 °C for 48 h.

#### Preparation of the Pd^0^@ magnetic amine-Functionalized UiO-66-NH_2_

A round bottom flask was charged by the magnetic UiO-66-NH_2_ (1.0 g) and dry THF (20 ml). Then, 2,4,6-trichloro-1,3,5-triazine (TCT: 10 mmol) at 0 °C with stirring bar for 7 h. Afterwards, the 14 mmol of K_2_CO_3_ and 5-phenyl tetrazole (20 mmol) was added to flask and stirred at room temperature. After 4h, the flask was equipped with condenser and refluxed at 50 °C for 24h. the final solid sample was separated and washed with water/methanol three times and dried in a vacuum oven at 60 °C for 12 h. In the end, the 0.2 g of final support was added to the saturated K_2_PdCl_4_ solution and stirred at room temperature for 24 h and then, Pd (II) was reduced to Pd (0) with aim of NaBH_4_ (15 mg). The Pd-catalyst was separated easily by an external magnet, washed with water/methanol three times and dried under reduced pressure. Based on ICP-OES analysis, the loading of Pd^0^ was found 0.78 mmol g^−1^ for fresh catalyst.

#### Preparation of the Pd^0^@ UiO-66-NH_2_

A round bottom flask was charged by the magnetic UiO-66-NH_2_ (0.2 g) and saturated K_2_PdCl_4_ solution and then stirred at room temperature for 24 h. In the end, Pd (II) was reduced to Pd (0) with aim of NaBH_4_ (15 mg). The Pd^0^@UiO-66-NH_2_ was separated easily by an external magnet, washed with water/methanol three times and dried under reduced pressure. Based on ICP-OES analysis, the loading of Pd^0^ was found 0.51 mmol g^−1^ for fresh catalyst.

### Catalytic performance for cyanation

The experiments were performed in a vessel containing aryl halide (1.0 mmol), potassium hexacyanoferrate(II) (0.2 mmol), potassium hydroxide (1.2 mmol), NMP (2.0 ml) and MOF-based catalyst (2.0 mol). The vessel was equipped with stirrer bar and temperature was increased from room temperature to 100 °C slowly. The reaction was monitored until completed (TLC, EtOAc: n-hexane, 1:5). Then, the mixture was diluted by EtOAc and water. The organic phase was with brine and dried with Na_2_SO_4_. The organic layers were mixed, purified by column chromatography, and confirmed by ^1^HNMR and ^13^CNMR.

### Catalytic performance for O-arylation

The experiments were performed in a vessel containing aryl halide (1.0 mmol), phenol (1.0 mmol), potassium hydroxide (1.2 mmol), H_2_O (2.0 ml) and MOF-based catalyst (2.0 mol). The vessel was equipped with stirrer bar and temperature was increased from room temperature to 80 °C slowly. The reaction was monitored until completed (TLC, EtOAc: n-hexane, 1:10). Then, the mixture was diluted by EtOAc and water. The organic phase was with brine and dried with Na_2_SO_4_. The organic layers were mixed, purified by column chromatography, and confirmed by ^1^HNMR and ^13^CNMR.

## Supplementary Information

**Supplementary Information.**

## References

[1] Zhao S-N, Wang G, Poelman D, Van Der Voort P (2018). Metal organic frameworks based materials for heterogeneous photocatalysis. Molecules.

[2] Chen G-J, Ma H-C, Xin W-L, Li X-B, Jin F-Z, Wang J-S, Liu M-Y, Dong Y-B (2017). Dual heterogeneous catalyst Pd–Au@ Mn (II)-MOF for one-pot tandem synthesis of imines from alcohols and amines. Inorg. Chem..

[3] Rowsell JL, Millward AR, Park KS, Yaghi OM (2004). Hydrogen sorption in functionalized metal−organic frameworks. J. Am. Chem. Soc..

[4] Couck S, Denayer JF, Baron GV, Rémy T, Gascon J, Kapteijn F (2009). An amine-functionalized MIL-53 metal−organic framework with large separation power for CO_2_ and CH_4_. J. Am. Chem. Soc..

[5] Li X, Van Zeeland R, Maligal-Ganesh RV, Pei Y, Power G, Stanley L, Huang W (2016). Impact of linker engineering on the catalytic activity of metal–organic frameworks containing Pd (II)–bipyridine complexes. ACS Catal..

[6] He C, Liu D, Lin W (2015). Nanomedicine applications of hybrid nanomaterials built from metal–ligand coordination bonds: nanoscale metal–organic frameworks and nanoscale coordination polymers. Chem. Rev..

[7] Dhakshinamoorthy A, Li Z, Garcia H (2018). Catalysis and photocatalysis by metal organic frameworks. Chem. Soc. Rev..

[8] Feng X, Jena HS, Leus K, Wang G, Ouwehand J, Van Der Voort P (2018). l-proline modulated zirconium metal organic frameworks: Simple chiral catalysts for the aldol addition reaction. J. Catal..

[9] Yang Q, Xu Q, Jiang H-L (2017). Metal–organic frameworks meet metal nanoparticles: synergistic effect for enhanced catalysis. Chem. Soc. Rev..

[10] Li L, Li Z, Yang W, Huang Y, Huang G, Guan Q, Dong Y, Lu J, Yu S-H, Jiang H-L (2021). Integration of Pd nanoparticles with engineered pore walls in MOFs for enhanced catalysis. Chem.

[11] Chen D, Yang W, Jiao L, Li L, Yu SH, Jiang HL (2020). Boosting catalysis of Pd nanoparticles in MOFs by pore wall engineering: the roles of electron transfer and adsorption energy. Adv. Mater..

[12] Lu G, Li S, Guo Z, Farha OK, Hauser BG, Qi X, Wang Y, Wang X, Han S, Liu X (2012). Imparting functionality to a metal–organic framework material by controlled nanoparticle encapsulation. Nat. Chem..

[13] Liu H, Chang L, Bai C, Chen L, Luque R, Li Y (2016). Controllable encapsulation of “clean” metal clusters within MOFs through kinetic modulation: towards advanced heterogeneous nanocatalysts. Angewandte.

[14] Chen Y-Z, Gu B, Uchida T, Liu J, Liu X, Ye B-J, Xu Q, Jiang H-L (2019). Location determination of metal nanoparticles relative to a metal–organic framework. Nat. Commun..

[15] Choi KM, Na K, Somorjai GA, Yaghi OM (2015). Chemical environment control and enhanced catalytic performance of platinum nanoparticles embedded in nanocrystalline metal–organic frameworks. J. Am. Chem. Soc..

[16] Abid HR, Tian H, Ang H-M, Tade MO, Buckley CE, Wang S (2012). Nanosize Zr-metal organic framework (UiO-66) for hydrogen and carbon dioxide storage. Chem. Eng. J..

[17] Lee DT, Zhao J, Oldham CJ, Peterson GW, Parsons GN (2017). UiO-66-NH_2_ metal–organic framework (MOF) nucleation on TiO_2_, ZnO, and Al_2_O_3_ atomic layer deposition-treated polymer fibers: role of metal oxide on MOF growth and catalytic hydrolysis of chemical warfare agent simulants. ACS Appl. Mater. Interfaces.

[18] Chen Z, Islamoglu T, Farha OK (2019). Toward base heterogenization: a zirconium metal–organic framework/dendrimer or polymer mixture for rapid hydrolysis of a nerve-agent simulant. ACS Appl. Nano Mater..

[19] Li X, Guo Z, Xiao C, Goh TW, Tesfagaber D, Huang W (2014). Tandem catalysis by palladium nanoclusters encapsulated in metal–organic frameworks. ACS Catal..

[20] Kardanpour R, Tangestaninejad S, Mirkhani V, Moghadam M, Mohammadpoor-Baltork I, Khosropour AR, Zadehahmadi F (2014). Highly dispersed palladium nanoparticles supported on amino functionalized metal–organic frameworks as an efficient and reusable catalyst for Suzuki cross-coupling reaction. J. Organomet. Chem..

[21] Yang Y, Yao H-F, Xi F-G, Gao E-Q (2014). Amino-functionalized Zr (IV) metal–organic framework as bifunctional acid–base catalyst for Knoevenagel condensation. J. Mol. Catal. A: Chem..

[22] Sun R, Liu B, Li BG, Jie S (2016). Palladium (II)@ zirconium-based mixed-linker metal–organic frameworks as highly efficient and recyclable catalysts for Suzuki and Heck cross-coupling reactions. ChemCatChem.

[23] Xiong G, Chen X-L, You L-X, Ren B-Y, Ding F, Dragutan I, Dragutan V, Sun Y-G (2018). La-metal–organic framework incorporating Fe3O4 nanoparticles, post-synthetically modified with Schiff base and Pd. A highly active, magnetically recoverable, recyclable catalyst for CC cross-couplings at low Pd loadings. J. Catal..

[24] Qin Y, Wang B, Li J, Wu X, Chen L (2019). Cobalt imine–pyridine–carbonyl complex functionalized metal–organic frameworks as catalysts for alkene epoxidation. Transit. Met. Chem..

[25] Hamzah HA, Gee WJ, Raithby PR, Teat SJ, Mahon MF, Burrows AD (2018). Post-synthetic mannich chemistry on metal–organic frameworks: system-specific reactivity and functionality-triggered dissolution. Chemistry.

[26] Chen J, Liu R, Guo Y, Chen L, Gao H (2015). Selective hydrogenation of biomass-based 5-hydroxymethylfurfural over catalyst of palladium immobilized on amine-functionalized metal–organic frameworks. ACS Catal..

[27] Chen H, Sun S, Liu YA, Liao X (2019). Nickel-catalyzed cyanation of aryl halides and hydrocyanation of alkynes via C–CN bond cleavage and cyano transfer. ACS Catal..

[28] Ueda Y, Tsujimoto N, Yurino T, Tsurugi H, Mashima K (2019). Nickel-catalyzed cyanation of aryl halides and triflates using acetonitrile via C–CN bond cleavage assisted by 1, 4-bis (trimethylsilyl)-2, 3, 5, 6-tetramethyl-1, 4-dihydropyrazine. Chem. Sci..

[29] Anbarasan P, Schareina T, Beller M (2011). Recent developments and perspectives in palladium-catalyzed cyanation of aryl halides: synthesis of benzonitriles. Chem. Soc. Rev..

[30] Damkaci F, Sigindere C, Sobiech T, Vik E, Malone J (2017). N-Picolinamides as ligands in Ullman type C–O coupling reactions. Tetrahedron Lett..

[31] Giri R, Brusoe A, Troshin K, Wang JY, Font M, Hartwig JF (2018). Mechanism of the Ullmann biaryl ether synthesis catalyzed by complexes of anionic ligands: evidence for the reaction of iodoarenes with ligated anionic CuI intermediates. J. Am. Chem. Soc..

[32] Ren Y, Wang W, Zhao S, Tian X, Wang J, Yin W, Cheng L (2009). Microwave-enhanced and ligand-free copper-catalyzed cyanation of aryl halides with K_4_[Fe (CN)_6_] in water. Tetrahedron Lett..

[33] Ren Y, Liu Z, Zhao S, Tian X, Wang J, Yin W, He S (2009). Ethylenediamine/Cu(OAc)_2_· H_2_O-catalyzed cyanation of aryl halides with K4 [Fe(CN)_6_]. Catal. Commun..

[34] Gholinejad M, Aminianfar A (2015). Palladium nanoparticles supported on magnetic copper ferrite nanoparticles: the synergistic effect of palladium and copper for cyanation of aryl halides with K4 [Fe (CN) 6]. J. Mol. Catal. A: Chem..

[35] Pourjavadi A, Keshavarzi N, Hosseini SH, Moghaddam FM (2018). Gold-Decorated 3D 2, 6-diaminopyridine network: a robust catalyst for the bromination of aromatic compounds. Ind. Eng. Chem. Res..

[36] Moghaddam FM, Ayati SE, Firouzi HR, Hosseini SH, Pourjavadi A (2017). Gold nanoparticles anchored onto the magnetic poly (ionic-liquid) polymer as robust and recoverable catalyst for reduction of Nitroarenes. Appl. Organomet. Chem..

[37] Ju Q, Luo W, Liu Y, Zhu H, Li R, Chen X (2010). Poly (acrylic acid)-capped lanthanide-doped BaFCl nanocrystals: synthesis and optical properties. Nanoscale.

[38] Xin F, Guo C, Chen Y, Zhang H, Qian L (2017). A novel triazine-rich polymer wrapped MMT: synthesis, characterization and its application in flame-retardant poly (butylene terephthalate). RSC Adv..

[39] Moghaddam FM, Saberi V, Kalvani P (2018). Phenyltetrazole as a new ligand for immobilization of palladium nanoparticles on SBA-15: a new robust catalyst with high loading of Pd for rapid oxidation and reduction. Chem. Sel..

[40] Ahmadipouya S, Haris MH, Ahmadijokani F, Jarahiyan A, Molavi H, Moghaddam FM, Rezakazemi M, Arjmand M (2021). Magnetic Fe_3_O_4_@ UiO-66 nanocomposite for rapid adsorption of organic dyes from aqueous solution. J. Mol. Liq..

[41] Min X, Yang W, Hui Y-F, Gao C-Y, Dang S, Sun Z-M (2017). Fe_3_O_4_@ ZIF-8: a magnetic nanocomposite for highly efficient UO^22+^ adsorption and selective UO^22+^/Ln^3+^ separation. Chem. Commun..

[42] Zhao H-X, Zou Q, Sun S-K, Yu C, Zhang X, Li R-J, Fu Y-Y (2016). Theranostic metal–organic framework core–shell composites for magnetic resonance imaging and drug delivery. Chem. Sci..

[43] Moghaddam FM, Jarahiyan A, Eslami M, Pourjavadi A (2020). A novel magnetic polyacrylonotrile-based palladium core−shell complex: a highly efficient catalyst for Synthesis of Diaryl ethers. J. Organomet. Chem..

