# Supplementary material for: An advancement in the synthesis of nano Pd@magnetic amine-Functionalized UiO-66-NH2 catalyst for cyanation and O-arylation reactions
Source: Sci Rep. 2021 May 31;11:11387. doi: 10.1038/s41598-021-90478-y (PMC8167105; doi:10.1038/s41598-021-90478-y)
Supplement: Supplementary file 1 — Supplementary Information. [file 41598_2021_90478_MOESM1_ESM.pdf]

Supporting Information

An Advancement in the Synthesis of Nano Pd@Magnetic Amine-Functionalized UiO-66-NH2 Catalyst for Cyanation and O-Arylation Reactions

Firouz Matloubi Moghaddam,[a]* Atefeh Jarahiyan,[a] Mahdi Heidarian Haris,[a] Ali Pourjavadi[b]

[a] Laboratory of Organic Synthesis and Natural Products, Department of Chemistry, Sharif University of Technology, Tehran, Iran, Email: matloubi@sharif.edu

[b] Polymer Research Laboratory, Department of Chemistry, Sharif University of Technology Tehran, Iran

Contents:

Experimantal section S3

NMR data of synthesized compounds...S4-S18

Experimental section

Materials

All chemicals were supplied from Merck Chemical Co and Sigma Aldrich which used without any additional purification.

Sample Characterization

FT–IR spectra were recorded by an ABB Bomem MB100 spectrometer at a range of 400–4000 cm-1 to depict the functional groups of samples with KBr pellets. 1H NMR and 13C NMR spectra were obtained on a Bruker Avance DRX-500 machine which CDCl3 and tetramethylsilane have been applied as a solvent and an internal standard at room temperature, respectively. The XRD patterns were accomplished on a PANalytical diffractometer with a copper target at 40 kV and 40 mA and Cu Kα (λ = 1.54 Å) for 2θ in the range of 0–80°. TGA results were investigated by taken on a STA503 TA instrument in the temperature range from room temperature to 800 °C heating rate of 20 °C min at nitrogen atmosphere. FE-SEM (TESCAN–Mira III at 15 KV) and TEM (Philips CM 120, 100 kV) were applied to investigate microstructure of the samples.

Data for Benzonitrile:

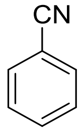

Colorless liquid; ^{1}H NMR (500 MHz, $CDCl_3$) δ 7.67 (d, *J* = 7.1 Hz, 2H), 7.63 (t, *J* = 7.5 Hz, 1H), 7.50 (t, *J* = 7.4 Hz, 2H); ^{13}C NMR (125 MHz, $CDCl_3$) δ 132.78, 132.00, 129.13, 118.75, 112.27.

Data for 2-Chlorobenzonitrile:

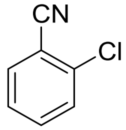

White solid; m.p 44-45 °C; ^{1}H NMR (500 MHz, $CDCl_3$) δ 7.65 (d, *J* = 5.4 Hz, 2H), 7.27 (dt, *J* = 17.3, 8.0 Hz, 2H); ^{13}C NMR (125 MHz, $CDCl_3$) δ 135.26, 135.19, 133.47, 129.98, 124.94, 116.32, 113.88.

Data for 4-Chlorobenzonitrile:

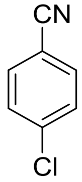

White solid; m.p 89-91 °C; ^{1}H NMR (500 MHz, $CDCl_3$) δ 7.63 (d, *J* = 7.5 Hz, 2H), 7.50 (d, *J* = 7.7 Hz, 2H); ^{13}C NMR (125 MHz, $CDCl_3$) δ 139.54, 133.37, 129.69, 117.92, 110.81.

Data for 2-Methylbenzonitrile:

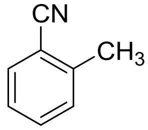

Yellow liquid; ^{1}H NMR (500 MHz, $CDCl_3$) δ 7.52-7.28 (m, 2H), 7.28-7.10 (m, 2H), 2.38 (s, 3H),; ^{13}C NMR (125 MHz, $CDCl_3$) δ 141.66, 132.60, 132.30, 126.21, 117.93, 112.63, 20.22.

Data for 3-Methylbenzonitrile:

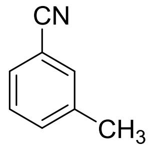

Yellow liquid; ^{1}H NMR (500 MHz, $CDCl_3$) δ 7.47 (s, 2H), 7.43 (d, *J* = 7.4 Hz, 1H), 7.37 (t, *J* = 7.6 Hz, 1H), 2.41 (s, 3H); ^{13}C NMR (125 MHz, $CDCl_3$) δ 138.94, 133.62, 132.34, 129.14, 118.90, 112.18, 21.00.

Data for 4-Methylbenzonitrile:

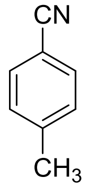

Colorless oil; ^{1}H NMR (500 MHz, $CDCl_3$) δ 7.56 (d, *J* = 7.5 Hz, 2H), 7.29 (d, *J* = 7.1 Hz, 2H), 2.44 (s, 3H); ^{13}C NMR (125 MHz, $CDCl_3$) δ 143.42, 132.01, 129.82, 119.09, 109.09, 21.78.

Data for 2-Aminobenzonitrile:

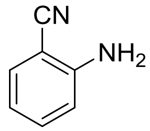

Yellow solid; m.p 47-48 °C; ^{1}H NMR (500 MHz, $CDCl_3$) δ 7.37 (dd, *J* = 7.5, 3.0 Hz, 1H), 7.36-7.29 (m, 1H), 6.80-6.67 (m, 2H), 4.49 (s, 2H); ^{13}C NMR (125 MHz, $CDCl_3$) δ 149.82, 134.04, 132.32, 117.91, 117.75, 115.26, 95.86.

Data for 3-Nitrobenzonitrile:

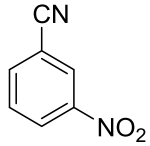

Yellow solid; m.p 115-117 °C; ^{1}H NMR (500 MHz, $CDCl_3$) δ 8.56 (s, 1H), 8.51 (d, *J* = 7.5, 1H), 8.03 (d, *J* = 6.9, 1H), 7.77 (t, *J* = 7.7, 1H); ^{13}C NMR (125 MHz, $CDCl_3$) δ 148.19, 137.78, 130.72, 127.54, 127.21, 116.64, 114.09.

Data for 4-Nitrobenzonitrile:

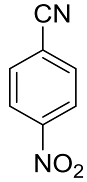

Yellow solid; m.p 149–150 °C; ^{1}H NMR (500 MHz, $CDCl_3$) δ 8.38 (d, *J* = 8.1, 2H), 7.92 (d, *J* = 8.0, 2H); ^{13}C NMR (125 MHz, $CDCl_3$) δ 150.05, 133.45, 124.27, 118.36, 116.74.

Data for 4-Bromobenzonitrile:

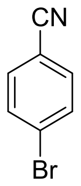

Light yellow solid; m.p 112-115 °C; ^{1}H NMR (500 MHz, $CDCl_3$) δ 7.66 (d, *J* = 8.2, 2H), 7.55 (d, *J* = 8.0, 2H); ^{13}C NMR (125 MHz, $CDCl_3$) δ 133.40, 132.63, 127.98, 118.00, 111.28.

Data for 4-Hydroxybenzonitrile:

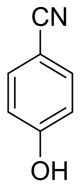

White solid; m.p 110-112 °C; ^{1}H NMR (500 MHz, $CDCl_3$) δ 7.58 (d, *J* = 7.2, 2H), 6.96 (d, *J* = 7.3, 2H), 9.41 (s, 1H); ^{13}C NMR (125 MHz, $CDCl_3$) δ 160.18, 134.33, 119.21, 116.48, 103.13.

Data for Picolinonitrile:

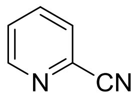

Light yellow oil; ^{1}H NMR (500 MHz, $CDCl_3$) δ 8.75 (s, 1H), 7.87 (t, *J* = 7.4, 1H), 7.73 (d, *J* = 7.2, 1H), 7.56 (s, 1H); ^{13}C NMR (125 MHz, $CDCl_3$) δ 151.11, 137.08, 133.95, 128.52, 126.97, 117.19.

Data for Isonicotinonitrile:

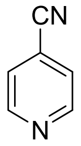

Yellow oil; ^{1}H NMR (500 MHz, $CDCl_3$) δ 8.84 (d, *J* = 3.1, 2H), 7.56 (d, *J* = 3.1, 2H); ^{13}C NMR (125 MHz, $CDCl_3$) δ 150.74, 125.21, 120.39, 116.34.

Data for 4-Acetylbenzonitrile:

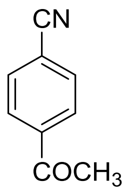

Pale yellow solid; m.p 47–49 °C; ^{1}H NMR (500 MHz, $CDCl_3$) δ 7.81 (d, *J*= 8.0 Hz, 1H), 7.64 (dd, *J*= 1.2, 7.5 Hz, 1H), 7.56 (dt, *J*= 1.2, 7.6 Hz, 1H), 7.50 (dt, *J*= 1.2, 7.6Hz, 1H), 2.52 (s, 3H); ^{13}C NMR (125 MHz, $CDCl_3$) δ 195.91, 138.88, 135.12, 133.05, 132.92, 130.08, 118.27, 110.59, 24.61.

Data for diphenyl ether:

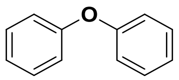

Colorless liquid; ^{1}H NMR (500 MHz, $CDCl_3$) δ 7.22 (d, *J* = 8.0 Hz, 2H), 7.27 (td, *J* = 7.0 Hz, 1H), 7.50 (t, *J* = 7.5 Hz, 2H); ^{13}C NMR (125 MHz, $CDCl_3$) δ 157.4, 129.9, 123.4, 119.0.

Data for 4-phenoxybenzonitrile:

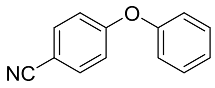

Oil; ^{1}H NMR (500 MHz, $CDCl_3$) δ 7.03 (d, *J* = 7.5 Hz, 2H), 7.09 (d, *J* = 7.5 Hz, 2H), 7.27 (t, *J* = 7.0 Hz, 1H), 7.44 (t, *J* = 7.5 Hz, 2H), 7.62 (d, *J* = 8.0 Hz, 2H); ^{13}C NMR (125 MHz, $CDCl_3$) δ 161.6, 154.9, 134.1, 130.2, 125.1, 120.4, 118.8, 117.9, 105.7.

Data for 4-Methyl-diphenylether:

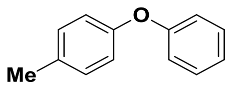

Colorless liquid; ^{1}HNMR ($CDCl_3$, 400 MHz) d: 2.41 (s, 3H), 7.00 (d, J = 8.0 Hz, 2H), 7.03–7.3 (m, 2H), 7.10–7.17 (m, 1H),7.24 (d, J = 8.4 Hz, 2H), 7.35 (t, J = 8.4 Hz, 2H); ^{13}CNMR ($CDCl_3$, 100 MHz) d: 20.4, 118.3, 119.6, 122.8,129.8, 130.1, 133.0, 154.2, 157.9.

Data for 4-Methoxy-diphenylether:

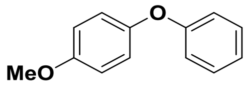

Colorless oil; 1H NMR (500 MHz, $CDCl_3$) ; δ 7.30 (t, J=7.5 Hz, 2H), 7.05–6.85 (m, 7H), 3.81 (s, 3H); ^{13}C NMR ($CDCl_3$, 125 MHz) δ 158.6, 156.0, 150.2, 129.7, 122.5, 121.0, 117.7, 115.0, 55.7.

Data for 1-nitro-4-phenoxybenzene: 4-Nitrophenyl phenyl ether

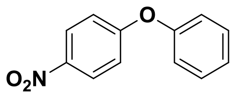

Yellow solid; m.p 57-59 °C; ^{1}H NMR (500 MHz, DMSO-d_6) δ 7.13 (d, *J* = 9.0 Hz, 2H), 7.21 (d, *J* = 7.5 Hz, 2H), 7.32 (t, *J* = 7.5 Hz, 1H), 7.51 (t, *J* = 7.5 Hz, 2H), 8.26 (d, *J* = 9.5 Hz, 2H); ^{13}C NMR (125 MHz, DMSO-d_6) δ 163.7, 155.0, 143.0, 131.4, 127.0, 126.4, 121.3, 118.1.

Data for 1-nitro-2-phenoxybenzene:

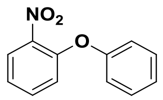

White solid; m.p 50-52 °C; ^{1}H NMR (500 MHz, $CDCl_3$) δ 7.03 (d, *J* = 7.5 Hz, 1H), 7.07 (d, *J* = 8.0 Hz, 2H), 7.21 (m, 2H), 7.40 (t, *J* = 8.5 Hz, 2H), 7.52 (td, *J* = 8.5 Hz, 1H), 7.97 (dd, *J* = 7 Hz, 1H); ^{13}C NMR (125 MHz, $CDCl_3$) δ 155.7, 153.7, 141.3, 134.1, 130.0, 125.7, 124.4, 123.1, 120.4, 119.0.

Data for 1-methoxy-4-(p-tolyloxy)benzene:

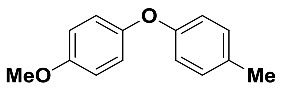

White solid; m.p 51-52 °C; ^{1}H NMR (500 MHz, $CDCl_3$) δ 2.34 (s, 3H), 3.82 (s, 3H), 6.87 (d, *J* = 6.5 Hz, 2H), 6.89 (d, *J* = 7.0 Hz, 2H), 6.98 (d, *J* = 9.5 Hz, 2H), 7.13 (d, *J* = 7.0 Hz, 2H); ^{13}C NMR (125 MHz, $CDCl_3$) δ 156.1, 155.6, 150.7, 132.0, 130.1, 120.3, 117.8, 114.8, 55.6, 20.6.

Data for 1-methoxy-4-(p-tolyloxy)benzene:

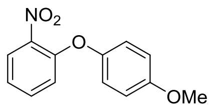

Off-white solid; m.p 97-99 °C; ^{1}H NMR (500 MHz, $CDCl_3$) δ 3.83 (s, 3H), 6.93 (d, *J* = 9.0 Hz, 3H), 7.04 (d, *J* = 9.0 Hz, 2H), 7.14 (t, *J* = 8.0 Hz, 1H), 7.47 (t, *J* = 7.5 Hz, 1H), 7.93 (d, *J* = 8.0 Hz, 1H); ^{13}C NMR (125 MHz, $CDCl_3$) δ 154.9, 149.9, 149.3, 139.3, 134.6, 126.1, 123.1, 119.8, 117.5, 114.3, 55.6.

Data for 4,4'-oxybis(methoxybenzene):

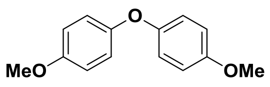

Yellow oil; ^{1}H NMR (500 MHz, $CDCl_3$) δ 6.95 (d, *J* = 9.0 Hz, 2H), 6.88 (d, *J* = 9.0 Hz, 2H), 3.81 (s, 3H); ^{13}C NMR (125 MHz, $CDCl_3$) δ 155.7, 151.9, 119.9, 115.1, 56.0.

Data for 1-phenoxynaphthalene:

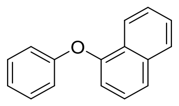

Off-White solid; m.p 51-53 °C; ^{1}H NMR (500 MHz, $CDCl_3$) δ 6.97 (d, *J* = 7.5 Hz, 1H), 7.06 (d, *J* = 8.0 Hz, 2H), 7.13 (t, *J* = 7.5 Hz, 1H), 7.36 (t, *J* = 7.5 Hz, 2H), 7.40 (t, *J* = 8.0 Hz, 1H), 7.50 (t, *J* = 7.0 Hz, 1H), 7.54 (t, *J* = 7.0 Hz, 1H), 7.64 (d, *J* = 8.0 Hz, 1H), 7.89 (t, *J* = 8.0 Hz, 1H), 8.2 (t, *J* = 7.5 Hz, 1H); ^{13}C NMR (125 MHz, $CDCl_3$) δ 157.2, 150.5, 134.8, 129.1, 127.7, 127.0, 126.6, 126.3, 123.1, 122.0, 121. 5, 119.0, 109.6.

Data for 2-phenoxynaphthalene:

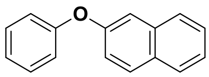

Off-White solid; m.p 48-49 °C; ^{1}H NMR (500 MHz, $CDCl_3$) δ 7.12 (d, *J* = 7.5 Hz, 2H), 7.18 (d, *J* = 7.5 Hz, 1H), 7.31 (dd, *J* = 8.5 Hz, 1H), 7.41 (m, 4H), 7.49 (t, *J* = 8.0 Hz, 1H), 7.74 (d, *J* = 8.0 Hz, 1H), 7.86 (t, *J* = 9.0 Hz, 1H); ^{13}C NMR (125 MHz, $CDCl_3$) δ 157.5, 155.4, 134.2, 130.5, 130,2, 130.2, 128.1, 127.5, 126.9, 125.1, 123.8, 120.3, 119.5, 114.4.

Data for the 4-(naphthalen-2-yloxy)benzonitrile:

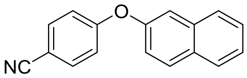

Yellow solid; m.p 104-106 °C; ^{1}H NMR (500 MHz, $CDCl_3$) δ 7.04 (d, *J* = 9.0 Hz, 2H), 7.17 (d, *J* = 7.5 Hz, 1H), 7.51 (m, 2H), 7.57 (t, *J* = 7.5 Hz, 1H), 7.61 (d, *J* = 7.5 Hz, 2H), 7.79 (d, *J* = 8.5 Hz, 1H), 7.95 (d, *J* = 8.0 Hz, 1H), 7.98 (t, *J* = 8.0 Hz, 1H); ^{13}C NMR (125 MHz, $CDCl_3$) δ 162.2, 150.4, 135.1, 134.2, 128.1, 126.9, 126.6, 125.8, 125.5, 121.6, 118.8, 117.5, 116.3, 105.7.

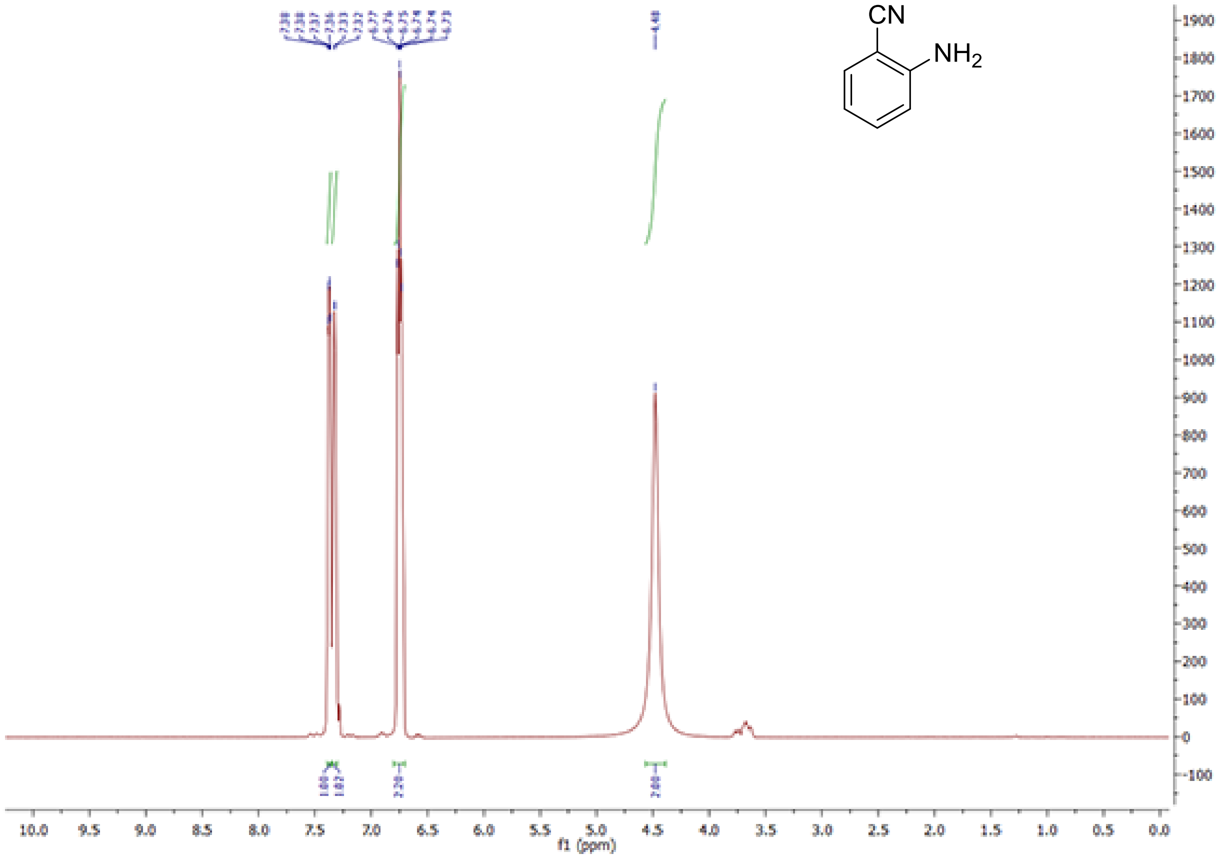

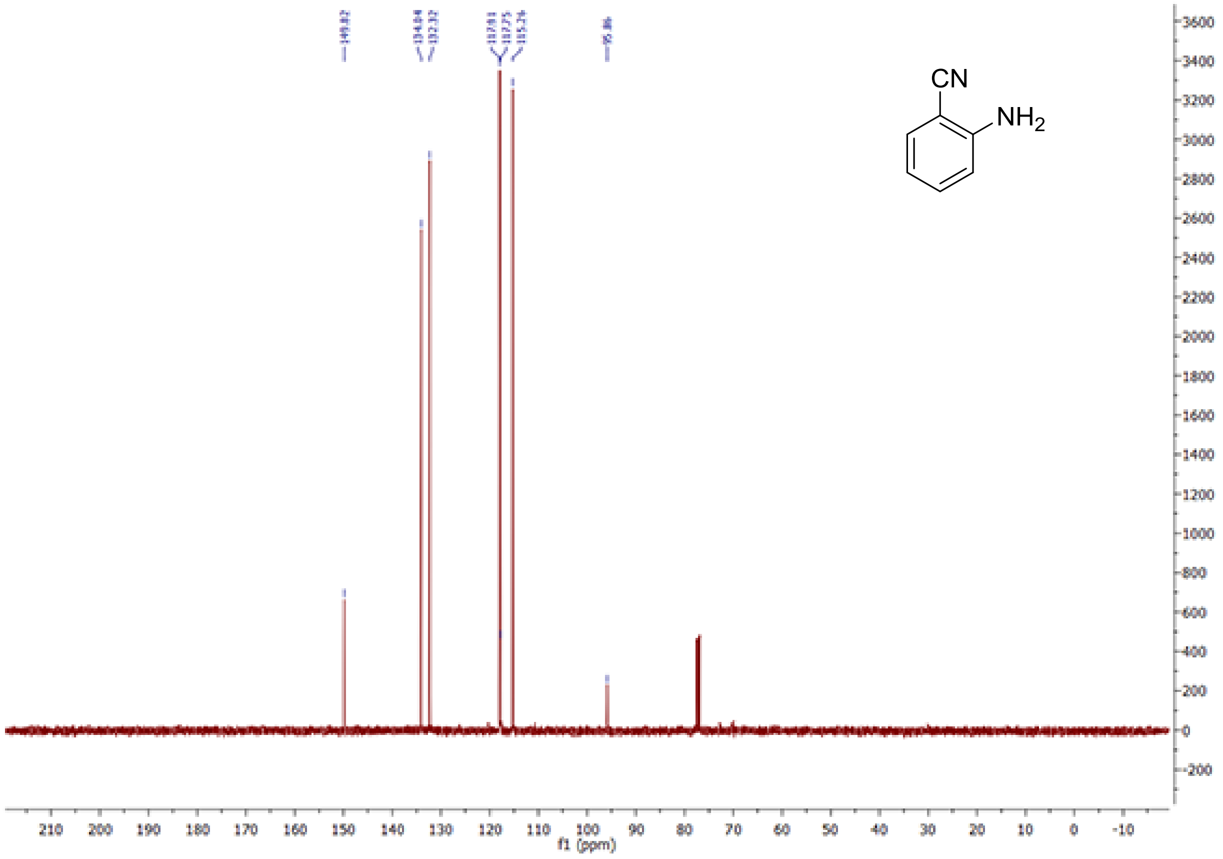

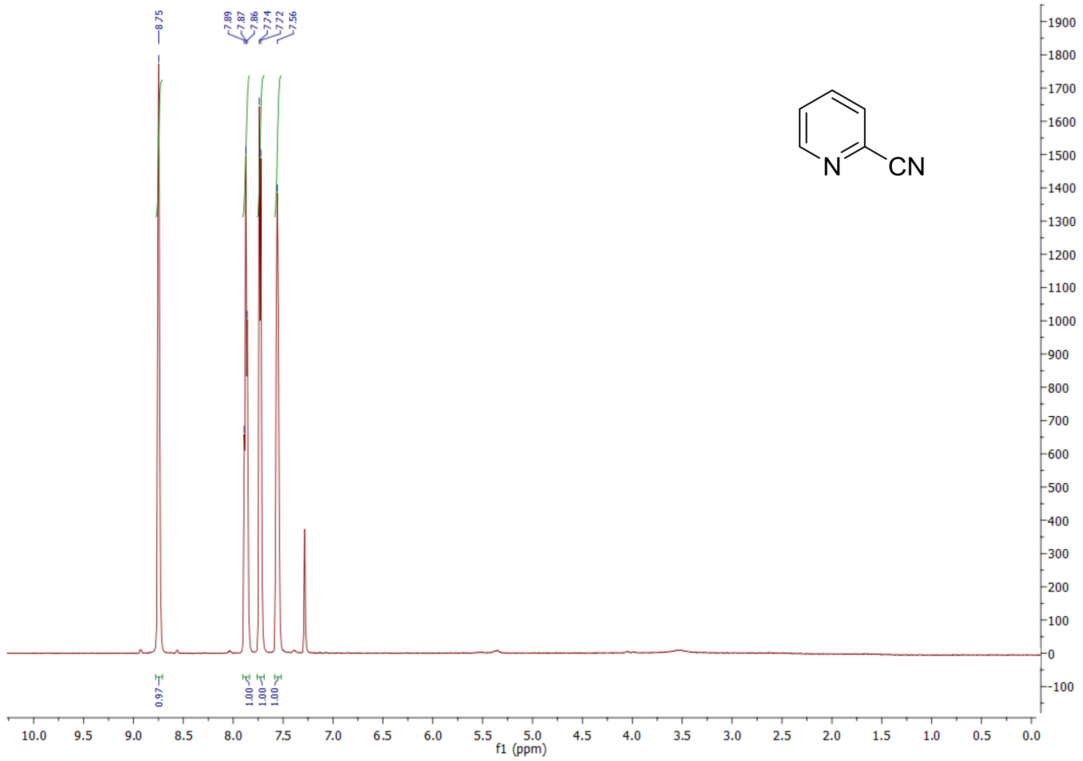
8.75
7.89
7.87
7.86
7.74
7.72
7.56
N
CN
0.97
1.00
1.00
1.00
f1 (ppm)

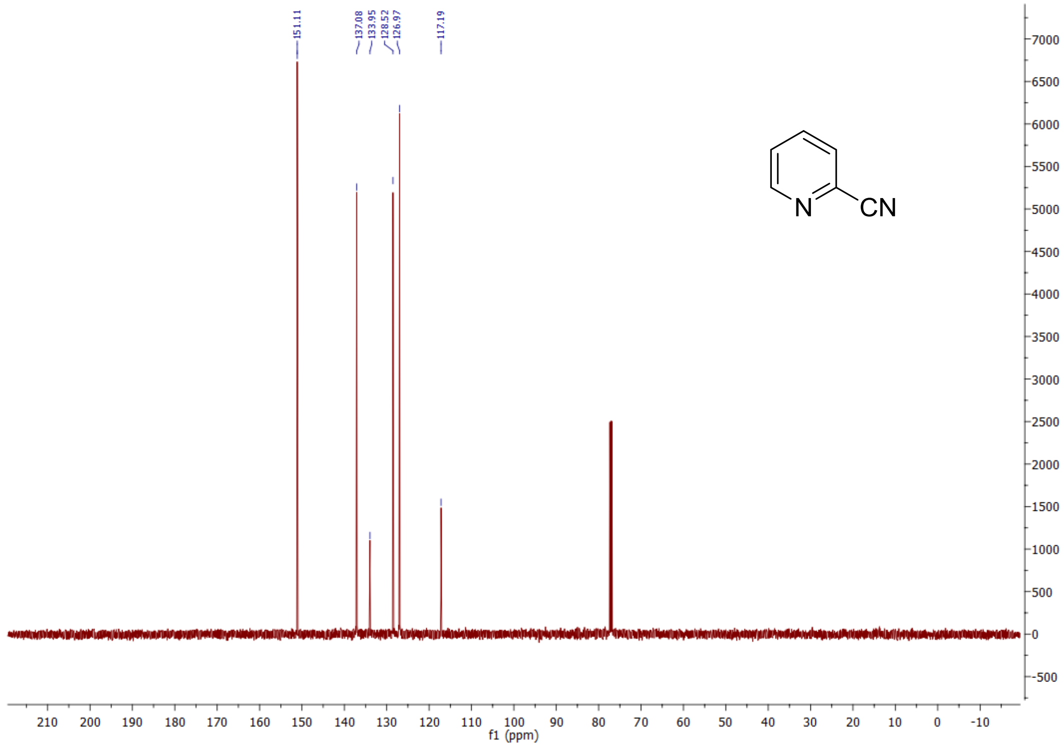
151.11
137.08
133.95
128.52
126.97
117.19
N
CN
f1 (ppm)

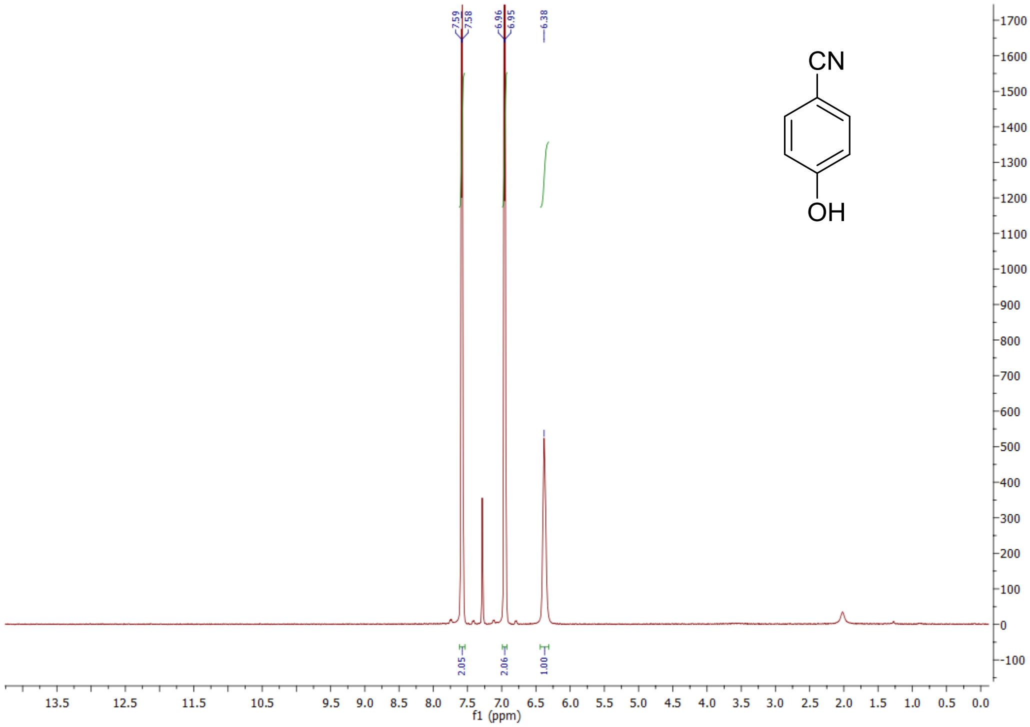
7.59
7.58
6.96
6.95
6.38
2.05
2.06
1.00
CN
OH
f1 (ppm)

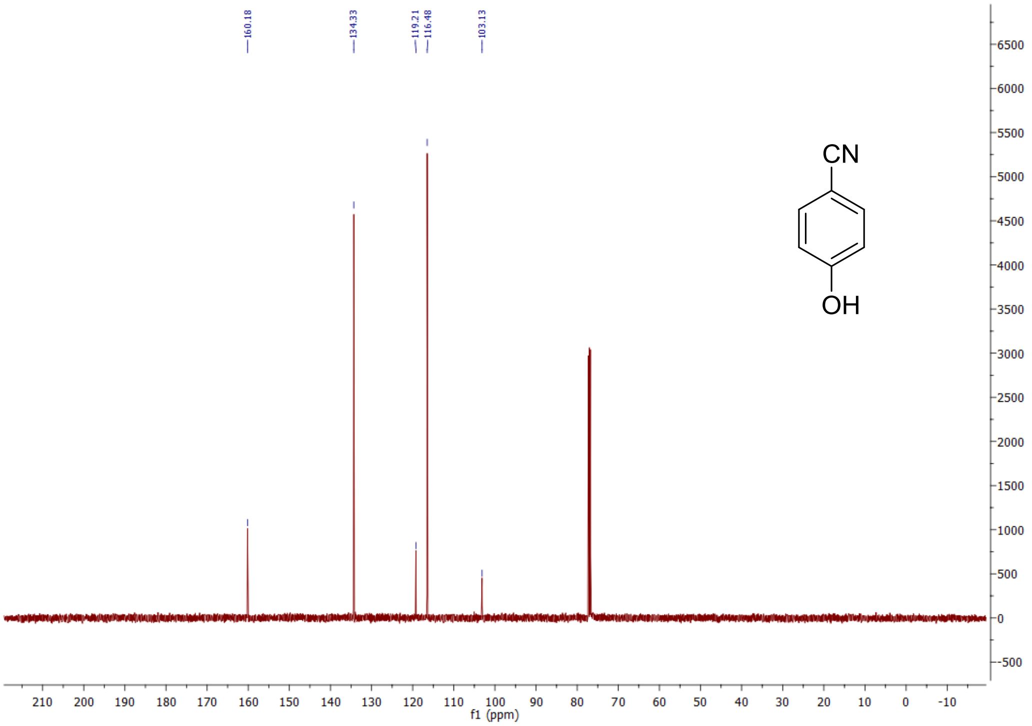
160.18
134.33
119.21
116.48
103.13
CN
OH
f1 (ppm)

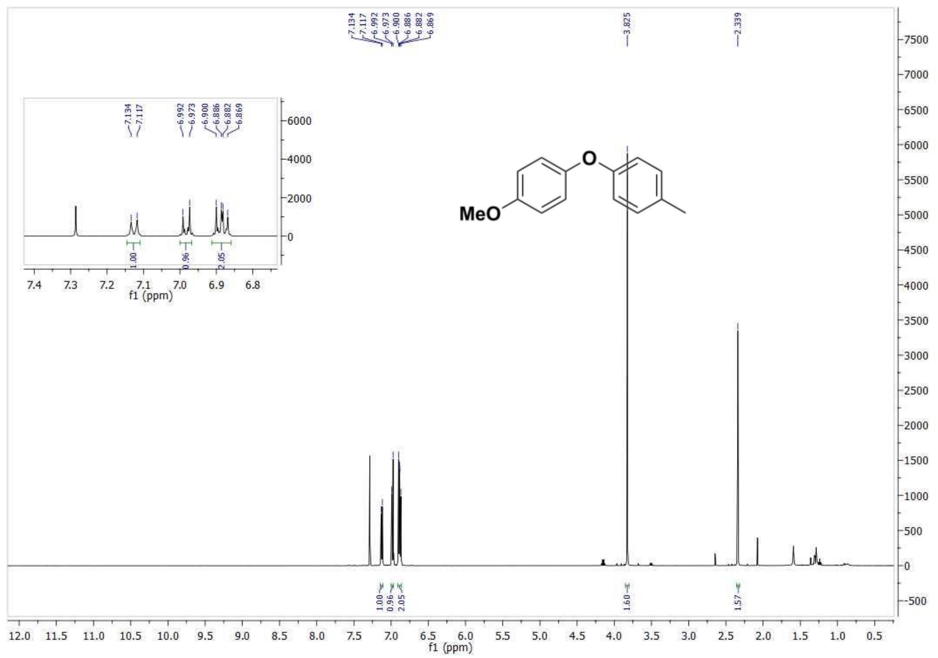

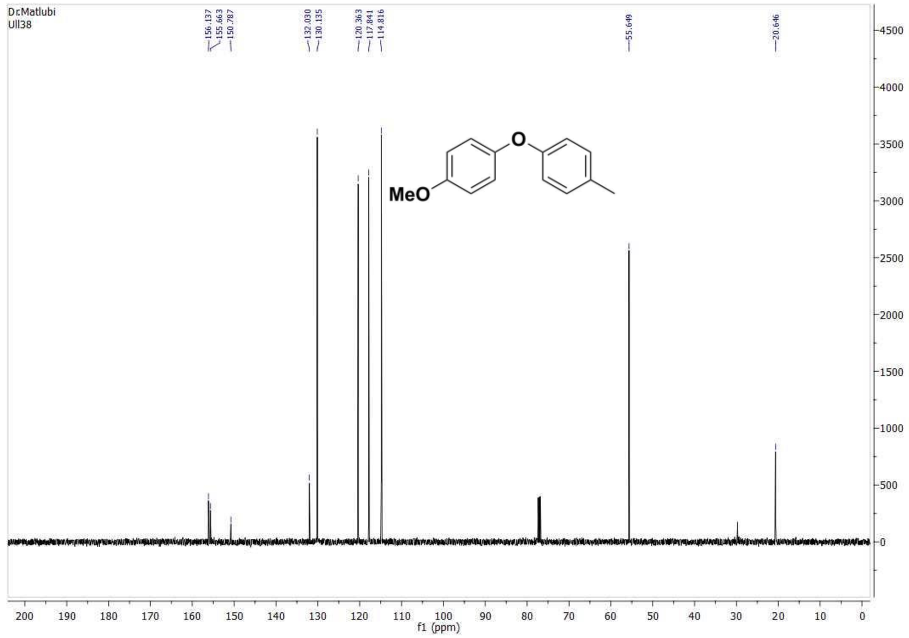

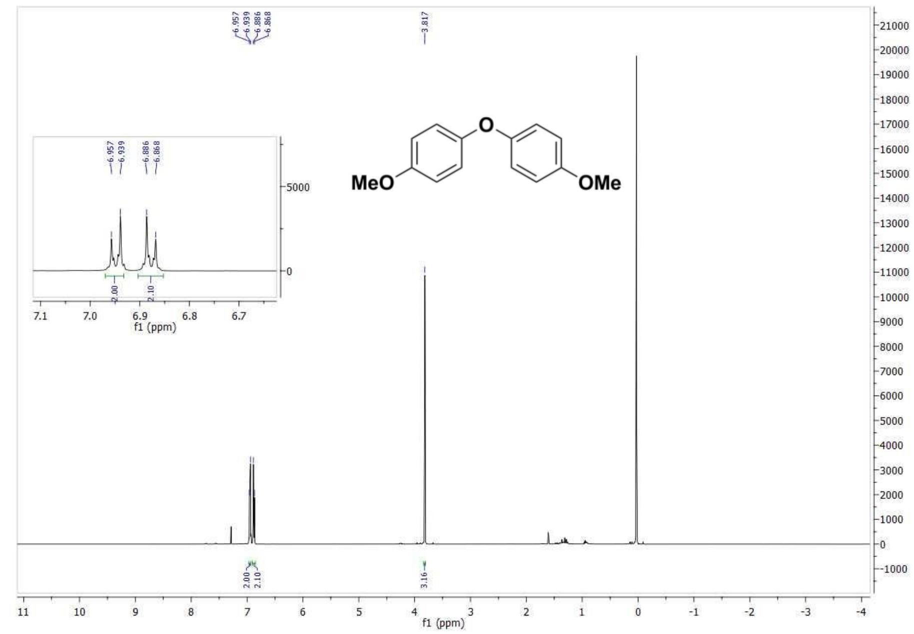
6.957
6.939
6.886
6.868
3.817
MeO
OMe
2.00
2.10
3.16
f1 (ppm)

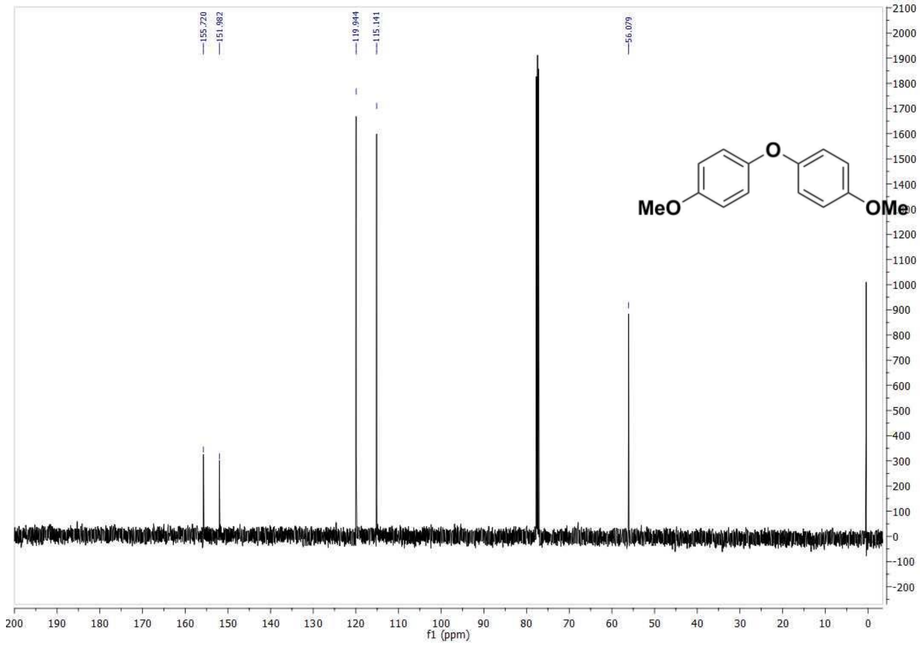
155.720
151.982
119.944
115.141
56.079
MeO
OMe
f1 (ppm)

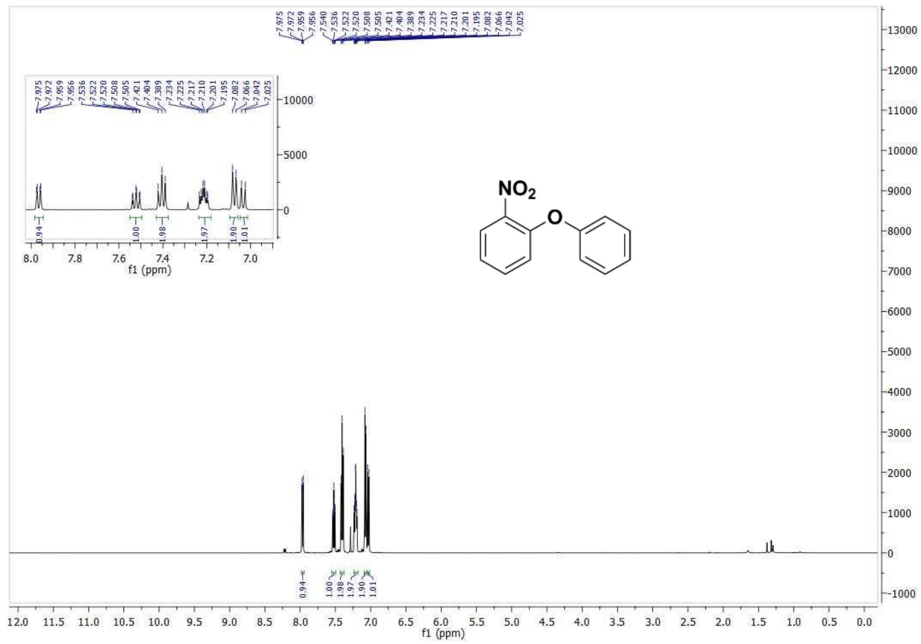
7.975 7.972 7.959 7.956 7.540 7.536 7.522 7.520 7.508 7.505 7.421 7.404 7.389 7.234 7.225 7.217 7.210 7.201 7.195 7.082 7.066 7.042 7.025
0.94 1.00 1.98 1.97 1.90 1.01
NO2
O
f1 (ppm)

1

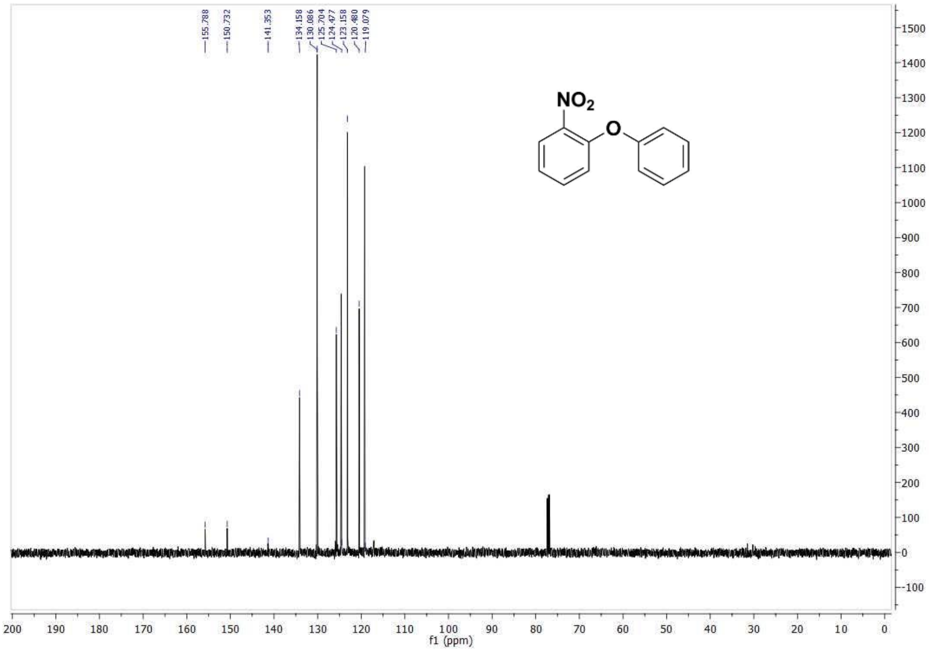
155.788 150.732 141.353 134.158 130.086 125.704 124.477 123.158 120.480 119.079
NO2
O
f1 (ppm)
